# Novel Adociaquinone Derivatives from the Indonesian Sponge *Xestospongia* sp.

**DOI:** 10.3390/md13052617

**Published:** 2015-04-28

**Authors:** Fei He, Linh H. Mai, Arlette Longeon, Brent R. Copp, Nadège Loaëc, Amandine Bescond, Laurent Meijer, Marie-Lise Bourguet-Kondracki

**Affiliations:** 1 Laboratoire Molécules de Communication et Adaptation des Micro-organismes, CNRS/MNHN 7245, Muséum National d’Histoire Naturelle, 57 rue Cuvier (C.P. 54), 75005 Paris, France; E-Mails: hefei@scsio.ac.cn (F.H.); mhoanglinh@yahoo.fr (L.H.M.); longeon@mnhn.fr (A.L.); 2 School of Chemical Sciences, University of Auckland, Private Bag 92019, Auckland 1142, New Zealand; E-Mail: b.copp@auckland.ac.nz; 3 ManRos Therapeutics, Perharidy Research Center, 29680 Roscoff, France; E-Mails: Nadege.Loaec@univ-brest.fr (N.L.); amandine.bescond@sb-roscoff.fr (A.B.); meijer@manros-therapeutics.com (L.M.)

**Keywords:** kinase inhibitor, adociaquinone, xestoadociaquinone, 14-carboxy-xestoquinol sulfate, xestoadociaminal, *Xestospongia* sp.

## Abstract

Seven new adociaquinone derivatives, xestoadociaquinones A (**1a**), B (**1b**), 14-carboxy-xestoquinol sulfate (**2**) and xestoadociaminals A–D (**3a**, **3c**, **4a**, **4c**), together with seven known compounds (**5**–**11**) were isolated from an Indonesian marine sponge *Xestospongia* sp. Their structures were elucidated by extensive 1D and 2D NMR and mass spectrometric data. All the compounds were evaluated for their potential inhibitory activity against eight different protein kinases involved in cell proliferation, cancer, diabetes and neurodegenerative disorders as well as for their antioxidant and antibacterial activities.

## 1. Introduction

Marine sponges of the genus *Xestospongia* have proved to be an extremely rich source of secondary metabolites with unprecedented molecular structures and various bioactivities [1,2,3]. Adocia- [4], halena- [5] and xesto-quinone [6] are the three main quinone-type skeletons identified from sponges of the genus *Xestospongia* sp. Among the most significant compounds, adociaquinones A and B, first isolated from the sponge *Adocia* sp. and then from the Philippine sponge *Xestospongia* sp. revealed inhibition of topoisomerase II in catalytic DNA unwinding and decatenation assays as well as inhibition of enzyme in the potassium sodium dodecyl sulfate KSDS assay [7]. Previous investigations on the South Pacific *Xestospongia* sp. by our group led to the isolation of a series of halenaquinone-type compounds, including xestosaprol C methylacetal, 3-ketoadociaquinones A and B, tetrahydrohalenaquinones A and B, halenaquinol sulfate, halenaquinone and orhalquinone [8]. Orhalquinone demonstrated significant inhibitory activities against both human and yeast farnesyltransferase enzymes, with IC_50_ values of 0.4 µM. 

**Figure 1:**
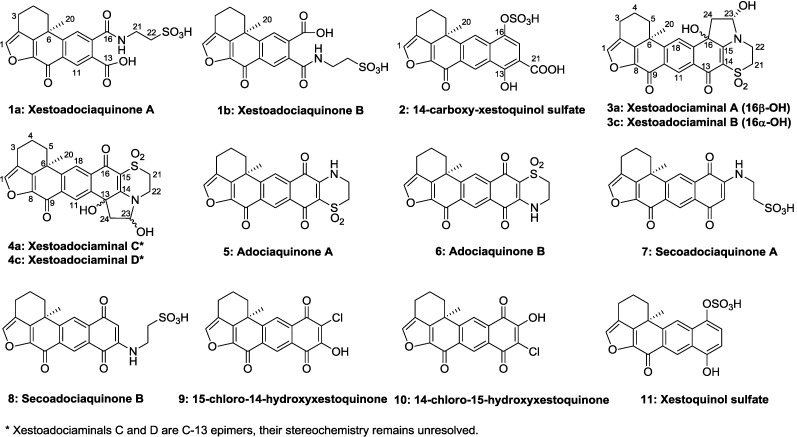
Structures of compounds **1**–**11** isolated from *Xestospongia* sp.

In our continuing search for bioactive compounds from marine sponges, we have chemically investigated the Indonesian sponge of *Xestospongia* sp. collected off North Sulawesi, the methanolic crude extract of which showed kinase inhibition, antimicrobial and antioxidant activities. Bio-guided fractionation of the extract led to the isolation of seven new adociaquinone derivatives **1a**–**4c**, together with seven known compounds, adociaquinone A **5** and B **6** [4], secoadociaquinones A **7** and B **8**, 15-chloro-14-hydroxyxestoquinone **9**, 14-chloro-15-hydroxyxestoquinone **10** [7] and xestoquinol sulfate **11** [9] (Figure 1). The known compounds were identified by comparison of their spectroscopic data with those of the literature. In this article, we describe the isolation and structural elucidation of the new compounds as well as their biological activities.

## 2. Results and Discussion

**Figure 2:**
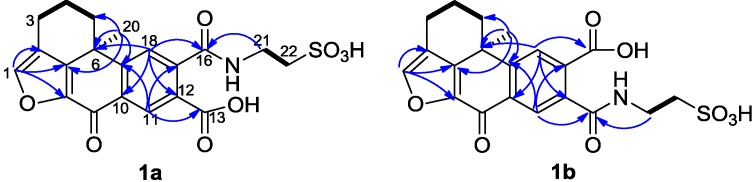
Selected ^1^H-^1^H COSY (−) and HMBC (^1^H→^13^C) correlations of **1a** and **1b**.

The mixture of **1a** and **1b** was isolated as a yellowish amorphous solid. The molecular formula was established as C_20_H_19_NO_8_S by the HRESIMS data (*m/z* 434.0905 [M + H]^+^) and indicates 14 degrees of unsaturation. The ^1^H and ^13^C NMR data (recorded in methanol-*d*_4_, Table 1) were nearly identical to the known compounds secoadociaquinones A and B [7], except for the loss of the quinonoid carbon resonances for C-14 and C-15. Furthermore, the key fragment ion peak at *m/z* 309 in ESI-MS spectrum, corresponding to loss of an -NH(CH_2_)_2_SO_3_H group, confirmed the presence of a taurine side chain in the molecule. In the ^1^H-^1^H COSY spectrum, we observed the correlations between the protons at δ_H_ 2.66, 2.89 (H_2_-3), δ_H_ 2.14, 2.32 (H_2_-4) and δ_H_ 1.67, 2.57 (H_2_-5), and between the protons at δ_H_ 3.84 (H_2_-21) and δ_H_ 3.16 (H_2_-22). The HMBC spectrum revealed the correlations between the proton at δ_H_ 7.70 (H-1) and the carbons at δ_C_ 123.2 (C-2), 145.2 (C-8) and 149.7 (C-7), and between the proton at δ_H_ 1.51 (H_3_-20) and the carbons at δ_C_ 32.3 (C-5), 38.1 (C-6), 149.7 (C-7) and 153.8 (C-19) (Figure 2). In addition, the HMBC correlations between the protons at δ_H_ 3.84 (H_2_-21) and the carboxyl carbons at 173.0 (**1a**) and 172.8 (**1b**) indicated the connection between the taurine side chain and a carboxyl group.

However, two sets of signals were observed in the ^1^H NMR spectrum around δ_H_ 7.70 and δ_H_ 8.64 with a ratio 2:3 suggesting the presence of two isomers. Compound **1a** showed resonances at δ_H_ 8.64 (*s*), 7.80 (*s*), and 7.70 (*s*), and the other set of resonances of compound **1b** were displayed at δ_H_ 8.37 (*s*), 7.97 (*s*), and 7.70 (*s*). In the HMBC spectrum, the proton at δ_H_ 7.80 (H-18 of compound **1a**) gave correlations with the carbons at δ_C_ 38.1 (C-6), 135.0 (C-12), 137.1 (C-10), and the carboxyl carbon at δ_C_ 173.0 (C-16). The signal at δ_H_ 8.64 (H-11) of compound **1a** showed HMBC correlations with δ_C_ 141.8 (C-17), 153.8 (C-19) and one carboxyl carbon at δ_C_ 172.7 (C-13). Similarly, the signal at δ_H_ 8.37 (H-11 of compound **1b**) correlated with the carbons at δ_C_ 141.8 (C-17), 154.2 (C-19) and the carboxyl carbon at δ_C_ 172.8 (C-13). The proton at δ_H_ 7.97 (H-18 of compound **1b**) showed correlations with the carbons at δ_C_ 38.1 (C-6), 134.5 (C-12), 137.1 (C-10) and 172.5 (C-16). These data confirmed that the structures of **1a** and **1b** were similar to 14, 15-*seco*adociaquinone skeleton.

In addition, correlations from the protons at δ_H_ 7.80 (H-18) and 3.84 (H_2_-21) to the carbon δ_C_ 173.0 (C-16) were observed in the HMBC spectrum of compound **1a**, while correlations from the protons at δ_H_ 8.37 (H-11) and 3.84 (H_2_-21) to the carbon δ_C_ 172.8 (C-13) were found in compound **1b**. Thus, the structures of compounds **1a** and **1b** were determined as presented in Figure 1 and named as xestoadociaquinone A (**1a**) and xestoadociaquinone B (**1b**).

**Table 1:** ^1^H and ^13^C NMR data for **1**–**4** (600 and 150 MHz, respectively) ^a^.

N°	1a ^b^		1b ^b^		2 ^c^		3a ^c^		3c ^c^		4a ^c^		4c ^c^	
	δ_C_, m	δ_H_, m ^d^	δ_C_, m	δ_H_, m ^d^	δ_C_, m	δ_H_, m ^d^	δ_C_, m	δ_H_, m ^d^	δ_C_, m	δ_H_, m ^d^	δ_C_, m	δ_H_, m ^d^	δ_C_, m	δ_H_, m ^d^
1	146.8	7.70, *s*	146.8	7.70, *s*	144.6	7.87, *s*	145.6	7.94, *dd*, 1.5, 2.0	145.5	7.93, *m*	145.7	7.95, *m*	145.7	7.95, *m*
2	123.2		123.3		121.2		121.4		121.5		122.8		122.7	
3	17.6	2.89, *m*	17.6	2.89, *m*	16.5	2.82, *m*	16.3	2.83, *m*	16.3	2.83, *m*	16.3	2.83, *m*	16.3	2.83, *m*
		2.66, *m*		2.66, *m*		2.58, *m*		2.59, *m*		2.59, *m*		2.59, *m*		2.59, *m*
4	19.5	2.32, *m*	19.5	2.32, *m*	18.1	2.20, *m*	17.8	2.20, *m*	17.8	2.20, *m*	17.9	2.21, *m*	17.9	2.21, *m*
		2.14, *m*		2.14, *m*		2.08, *m*		2.05, *m*		2.05, *m*		2.05, *m*		2.05, *m*
5	32.3	1.67, *m*	32.2	2.57, *m*	31.7	2.64, *m*	30.4	2.67, *m*	30.6		30.8	2.55, *m*	30.6	2.55, *m*
		2.57, *m*		1.67, *m*		1.68, *m*		1.56, *m*				1.58, *m*		1.58, *m*
6	38.1		38.1		35.8		36.7		36.5		36.4		36.4	
7	149.7		149.7		146.4		147.3		147.3		147.8		147.8	
8	145.2		145.2		143.9		143.1		143.2		143.3		143.2	
9	172.5		172.5		171.4		170.4		170.5		170.3		170.3	
10	137.1		137.1		128.8		142.6		142.7		135.1		135.2	
11	128.4	8.64, *s*	128.4	8.37, *s*	124.7	9.03, *s*	124.9	8.66, *s*	124.8	8.65, *s*	124.5	8.19, *s*	124.4	8.19, *s*
12	135.0		134.5		124.6		130.2		130.1		137.8		137.8	
13	172.7 ^e^		172.8 ^e^		162.1		173.6		173.6		72.4		72.5	
14					111.0				104.0		164.5		164.5	
15					121.7	7.75, *s*	164.2		164.3		104.2		104.3	
16	173.0		172.5		136.6		72.7		72.6		173.5		173.7	
17	141.8		141.8		132.2		132.9		132.9		133.9		134.2	
18	126.2	7.80, *s*	126.9	7.97, *s*	119.0	8.16, *s*	123.2	7.84, *s*	123.3	7.86, *s*	122.8	8.18, *s*	122.7	8.16, *s*
19	153.8		154.2		146.7		154.3		154.2		150.9		151.1	
20	32.6	1.51, *s*	32.5	1.52, *s*	34.4	1.44, *s*	31.8	1.43, *s*	31.6	1.48, *s*	32.0	1.43, *s*	32.0	1.43, *s*
21	37.2	3.84, *m*	37.2	3.84, *m*	171.8		48.7	3.38, *m*	48.7	3.93, *m*	48.7	3.39, *m*	48.7	3.39, *m*
								3.40, *m*		3.90, *m*		3.33, *m*		3.33, *m*
22	51.1	3.16, *m*	51.2	3.16, *m*			39.4	3.93, *m*	39.4	3.38, *m*	39.4	3.95, *m*	39.4	3.95, *m*
								3.90, *m*		3.34, *m*		3.89, *m*		3.89, *m*
23							87.5	5.61, *m*	87.4	5.61, *m*	87.5	5.62, *m*	87.5	5.62, *m*
24							44.1	3.24, *m*	44.1	3.24, *m*	44.0	3.19, *m*	44.0	3.19, *m*
								2.12, *m*		2.12, *m*		2.04, *m*		2.04, *m*
-OH-16/13								6.78, *d*, 2.0		6.88, *brs*		6.79, *d*, 1.0		6.74, *d*, 1.0
-OH-23								6.96, *d*, 9.0		6.99, *brs*		7.03, *d*, 8.5		7.03, *d*, 8.5

^a^ All the data were assigned by HSQC, HMBC, COSY and NOESY experiments; ^b^ Recorded in methanol-*d*_4_; ^c^ Recorded in DMSO-*d*_6_; ^d^ m: multiplicity, *J* in Hertz; ^e^ Not observed in DEPT spectrum, but only in HMBC spectrum.

**Figure 3:**
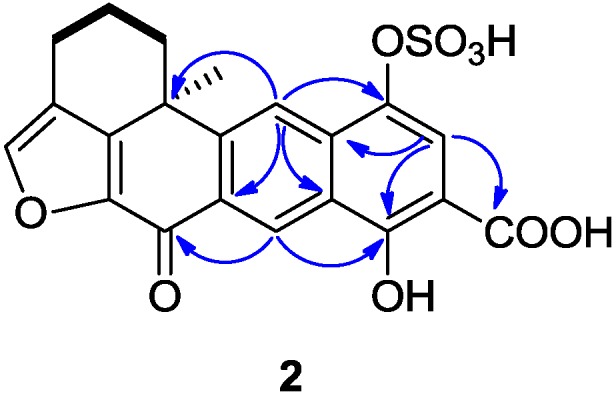
Selected ^1^H-^1^H COSY (−) and HMBC (^1^H→^13^C) correlations of **2**.

Compound **2** was isolated as a yellowish amorphous solid, with the molecular formula of C_21_H_16_O_9_S deduced from HR-ESIMS data (*m/z* 443.0447 [M + H]^+^), indicating 16 degrees of unsaturation. The ^1^H and ^13^C NMR data (recorded in DMSO-*d*_6_, Table 1) showed similarities with those of the xestoquinol skeleton of compound **11**. The major difference between compounds **2** and **11** [8] was the presence in the ^13^C NMR spectrum of one carboxyl carbon signal at δ_C_ 171.8. The position of a carboxyl group was determined thanks to the HMBC correlations between the proton at δ_H_ 7.75 (H-15) and the carbons at δ_C_ 132.2 (C-17), 162.1(C-13), and 171.8 (C-21) (Figure 3). Another major difference is the lack of the ortho-coupled protons system between H_14_-H_15_, replaced by the singlet due to H_15_. Therefore, the structure of **2** was determined as being 14-carboxy-xestoquinol sulfate.

**Figure 4:**
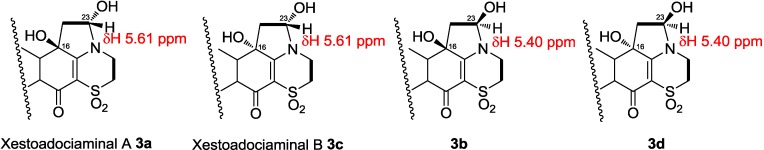
Relative configuration of the hemiaminal diastereomers **3a**–**d**.

The structure elucidation of xestoadociaminals A **3a** and B **3c** was performed on two fractions, each of which contained the two natural products, and their hemiaminal diastereomers, in differing proportions. Compound **3a** was obtained as a yellowish solid after being recrystallized slowly from a methanolic solution of **3a** and **3c**. ^1^H NMR rapidly established that the sample was comprised of **3a** and **3c** in a ratio of 1:0.14, in addition to two further minor compounds (hemiaminal diastereomers, see later) also being present, again in a relative ratio of 1:0.14 (see ^1^H NMR spectrum in Supplementary Information). Particularly diagnostic of the presence of four compounds, were ^1^H resonances at δ_H_ 7.84, 7.78, 7.86 and 7.81 (H-18, all singlets) and δ_H_ 8.66, 8.64, 8.65 and 8.62 (H-11, all singlets), with both sets of signals observed in relative ratios of 1:0.14:0.14:0.02, respectively. The molecular formula of **3a** was assigned as C_24_H_21_NO_7_S based on its HRESIMS data (*m/z* 468.1100 [M + H]^+^), indicating 17 degrees of unsaturation. The presence of hydroxyl and carbonyl functional groups was deduced from the bands at 3649, 1752, and 1717 cm^−1^ in the IR spectrum. The DEPT spectrum indicated 24 carbons, including one methyl, six methylenes, four methines, and thirteen quaternary carbons. The ^1^H and ^13^C NMR data (recorded in DMSO-*d*_6_, Table 1) of **3a** showed some similarities with adociaquinone A [6,7]. In the HMBC spectrum, the correlations between the proton at δ_H_ 8.66 (H-11) and the carbons at δ_C_ 132.9 (C-17), 154.3 (C-19), 170.4 (C-9) and 173.6 (C-13), between the proton at δ_H_ 7.84 (H-18) and the carbons at δ_C_ 36.7 (C-6), 130.2 (C-12), 142.6 (C-10), as well as between the proton at δ_H_ 7.94 (H-1) and the carbons at δ_C_ 121.4 (C-2), 143.1 (C-8), and 147.3 (C-7), confirmed the partial fragment of adociaquinone skeleton [5,7]. The main differences were the lack of one carbonyl group and the presence of additional signals corresponding to one oxygenated quaternary carbon [δ_C_ 72.7 (C-16)], one oxymethine [δ_C_ 87.5 (C-23)/δ_H_ 5.61 (1H, *m*, H-23)] and one methylene [δ_C_ 44.1 (C-24)/δ_H_ 3.24 (2H, *m*, H-24)] signal in the ^1^H and ^13^C NMR spectra of **3a**. From the ^1^H-^1^H COSY spectrum, we observed the correlations between the protons at δ_H_ 2.83–2.59 (H_2_-3) and the protons at δ_H_ 2.20–2.05 (H_2_-4), which in turn gave correlations with the protons at δ_H_ 2.67, 1.56 (H_2_-5), and between the proton at δ_H_ 3.93–3.90 (H_2_-22) and the protons at δ_H_ 3.38, 3.34 (H_2_-21), and particularly between the proton at δ_H_ 5.61 (H-23) and the protons at δ_H_ 3.24–2.12 (H_2_-24) and the exchangeable proton at δ_H_ 6.96 (OH-23). The presence of a pyrrolidine ring in compound **3a** was deduced by HMBC correlations between the proton at δ_H_ 3.24–2.12 (H_2_-24) and the carbons at δ_C_ 72.7 (C-16), 87.5 (C-23), and 164.2 (C-15), and between the proton at δ_H_ 5.61 (H-23) and the carbon at δ_C_ 44.1 (C-24). Furthermore, the HMBC correlations observed from the exchangeable proton at δ_H_ 6.78 (OH-16) and the proton at δ_H_ 7.84 (H-18) to the carbon at δ_C_ 72.7 (C-16), and from the other exchangeable proton at δ_H_ 6.96 (OH-23) to the carbon at δ_C_ 87.5 (C-23) (Figure 4) confirmed the presence of two hydroxyl groups at positions 16 and 23. Therefore, it was concluded that the structure of **3a** contained a pyrrolidine ring fused between the dioxothiazine and quinone rings of adociaquinone A. With two stereogenic centres being present in this new ring system (at C-16 and C-23), in addition to the chiral centre at C-6, the four compounds in the sample being studied were ascribed to being pairs of hemiaminal diastereomers (each present in a 1:0.14 relative ratio) associated with each of the two possible C-16 stereoisomers. Observation of a NOESY correlation between the hemiaminal proton at δ_H_ 5.61 (H-23) and the hydroxyl proton at δ_H_ 6.78 (OH-16), indicated the relative disposition between these two protons in the dominant diastereomer present in the sample, however a lack of other correlations prevented determination of configuration relative to the stereocentre at C-6. While many of the ^1^H and ^13^C resonances associated with the hemiaminal diastereomer of **3a** (**3b**) were co-incident with those of **3a**, discernible signals attributable to **3b** were observed at δ_H_ 8.64/δ_C_ 124.9 (CH-11), δ_H_ 7.78/δ_C_ 123.3 (CH-18), δ_H_ 5.40/δ_C_ 88.3 (CH-23) and δ_C_ 74.7 (C-16). With NMR resonances assigned for each of **3a**/**3b**, attention then turned to a second column fraction that also contained the same four sets of NMR signals. Integration of the ^1^H NMR spectrum of this second fraction indicated it to be enriched in a second compound **3c**, also present with its hemiaminal diastereomer **3d**, with relative ratios of 1:0.15:1:0.15 (**3a**:**3b**:**3c**:**3d**). Differences between ^1^H NMR data observed for **3c** compared to **3a** were associated with the resonance of the exchangeable proton at OH-16 (δ_H_ 6.78 for **3a** and δ_H_ 6.88 for **3c**), H-11 (8.66 *vs.* 8.65), H-18 (7.84 *vs.* 7.86) while ^13^C NMR shift differences were observed for C-16 (72.7 *vs.* 72.6), C-23 (87.45 *vs.* 87.40), and C-18 (123.1 *vs.* 123.3). It was thus concluded that compound **3c** was likely the C-16 epimer of **3a**. It was named xestoadociaminal B. The fourth very minor constituent of the fraction mixtures was then presumed to be the hemiaminal diastereomer of **3c** (Figure 4).

**Figure 5:**
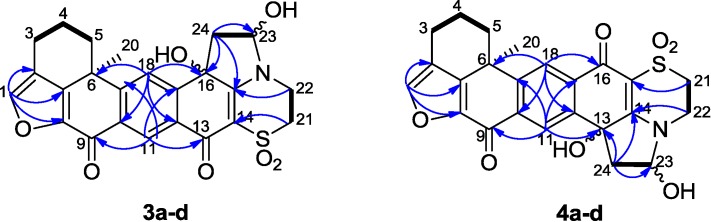
Selected ^1^H-^1^H COSY (−) and HMBC (^1^H→^13^C) correlations of compounds **3a**–**d** and **4a**–**d**.

Compound **4** was obtained as a 1:1:0.29:0.29 mixture of four diastereoisomers (see ^1^H NMR spectrum in supplementary data), isolated as a yellowish amorphous solid. The molecular formula was also assigned as C_24_H_21_NO_7_S, being isomeric with **3a**–**d**. The ^1^H and ^13^C NMR data (recorded in DMSO-*d*_6_, Table 1) were also similar to those observed for compounds **3a**–**d**. The partial fragment of an adociaquinone skeleton was confirmed by the HMBC correlations between the proton at δ_H_ 8.19 (H-11) and the carbons at δ_C_ 133.9 (C-17), 150.9 (C-19) and 170.3 (C-9), between the proton at δ_H_ 8.18 (H-18) and the carbons at δ_C_ 36.5 (C-6), 135.2 (C-10), 137.8 (C-12) and 173.5 (C-16), and between the proton at δ_H_ 7.95 (H-1) and the carbons at δ_C_ 122.8 (C-2), 143.3 (C-8) and 147.8 (C-7). However, in contrast to the case of compound **3a**, the HMBC spectrum also showed key correlations between the proton at δ_H_ 8.18 (H-18) and the carbon at δ_C_ 173.5 (C-16), and between the proton at δ_H_ 8.19 (H-11) and the carbon at δ_C_ 72.4 (C-13), between the exchangeable proton at δ_H_ 6.79 (OH-13) and the carbons at δ_C_ 44.1 (C-24), 72.4 (C-13), and 137.8 (C-12) and between the other exchangeable proton at δ_H_ 7.03 (OH-23) to the carbons at δ_C_ 44.1 (C-24) and 87.5 (C-23) (Figure 5). These correlations again identified the presence of a pyrrolidine ring fused between the dioxothiazine and quinone rings of an adociaquinone-type molecule, but in contrast to ring fusion to C-16 in **3a**–**d**, fusion in compound **4** was at C-13. Therefore, it was concluded that the fraction contained all four diastereomers represented by structures **4a**–**d** with the two major components **4a** and **4c** being C-13 epimers and the minor components **4b** and **4d** being their corresponding hemiaminal diastereomers. The major components were named xestoadociaminals C and D; their relative configurations remain unresolved due to overlapping signals. Biogenetically, the structures of **3a**–**d** and **4a**–**d** represent the addition of ethanal to adociaquinones A and B. However, it should be noted that ethanol was not used for the storage of the sponge nor in any chromatographic purification steps.

**Figure 6:**
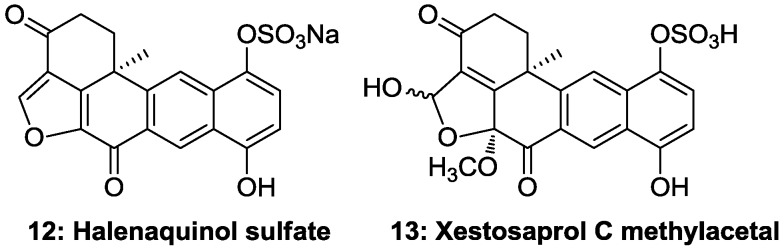
Halenaquinone derivatives of the marine sponge *Xestospongia testudinaria collected* in Solomon Islands.

All compounds were tested against eight different protein kinases relevant to cell proliferation, cancer, diabetes and neurodegenerative disorders along with the related compounds **12** and **13** (Figure 6), previously isolated from the marine sponge *Xestospongia* sp., [8] in order to establish structure-activity relationships. Compounds **6** and **8** revealed a modest but selective inhibitory activity towards CDK9/cyclin T (IC_50_: 3 µM) and CDK5/p25 (IC_50_: 6 µM), respectively. Compound **12**, which is a sodium derivative and differs from **11** by the presence of the ketone group in the position 3, showed significant activity against most protein kinases ranging from 0.5 to 7.5 µM (Table 2), while compound **13**, with a hydroxyl in position C-1 and a methoxyl at C-8, showed marginal activity against DYRK1A. This information suggests that the presence of a ketone group in position 3 and eventually a non-substituted furan ring are important for the activity. Therefore, adociaquinone derivatives could be of interest in the discovery of new potential kinase inhibitors.

All the compounds were also tested for potential antioxidant and antibacterial activities. Only compound **11** showed moderate antibacterial activity with an IC_50_ value of 125 μM against *Staphylococcus aureus*. 

**Table 2:** Protein kinase inhibitory activity of compounds **7**, **8**, **12** and **13**^ a^.

Compound ^b^	CDK1	CDK2	CDK5	CDK9	CK1	CLK1	DYRK1A	GSK3
**6**	>10	>10	>10	3	>10	>10	>10	>10
**8**	>10	>10	6	>10	>10	>10	>10	>10
**12**	4.3	7.5	2.2	0.5	5.2	0.71	0.61	0.61
**13**	>10	>10	>10	>10	>10	>10	9.3	>10

^a^ IC_50_ values, calculated from the dose-response curves, are reported in μM; ^b^ All other isolated compounds did not show any significant activity in this protein kinase panel at the highest concentration tested (IC_50_ > 10 μM).

## 3. Experimental Section 

### 3.1. General Experimental Procedures

Optical rotations were recorded on a Perkin-Elmer 341 polarimeter (Villebon-sur-Yvette, France). IR spectra were recorded on a FT-IR Shimadzu 8400 S spectrometer (Noisiel, France). UV spectra were recorded on a UVIKON 930 spectrometer (Kontron, France). Mass spectra were recorded on an API Q-STAR PULSAR I of Applied Biosystems (Concord, ON, Canada). NMR spectra were obtained on either a Bruker Avance 400 or 600 spectrometer (Wissenburg, France) using standard pulse sequences. The acquisition of HMBC spectra was optimized for either 7 or 8.3 Hz. Column chromatography (CC) purifications were performed using silica gel (200~400 mesh; Merck, Darmstadt, Germany) and Sephadex LH-20 (Amersham Pharmacia, Uppsala, Sweden). Fractions were analyzed by TLC using aluminum-backed sheets (Silica gel 60 F254) and visualized under UV (254 nm) and Lieberman spray reagent. Preparative TLC used glass plate coated with silica gel 60 F254, 0.25 mm thick (Merck, Darmstadt, Germany). Flash chromatography was carried out on Buchi C-615 pump system (Rungis, France). Analytical and semi-preparative reverse-phase (Gemini C6-phenyl, Luna C18 and HILIC, Phenomenex, Le Pecq, France) columns were performed with an Alliance HPLC apparatus (model 2695, Waters, Saint-Quentin en Yvelines, France), equipped with a photodiode array detector (model 2998, Waters), an evaporative light-scattering detector (model Sedex 80, Sedere, Alforville, France), and the Empower software.

### 3.2. Sponge Material

Specimens of *Xestospongia* sp. were collected in the North Sulawesi (Bunaken and other islands/reefs near Manado), Indonesia and were identified by Professor Van Soest, University of Amsterdam, the Netherlands.

### 3.3. Extraction and Isolation

Sponge specimens (500 g) were immediately immersed in MeOH after collection. The MeOH solution was evaporated and the aqueous residue was extracted and partitioned first with 1 L CH_2_Cl_2_ to give the CH_2_Cl_2_ extract (6.5 g). The aqueous phase was extracted with 1 L EtOAc and partitioned to afford to the EtOAc extract (1.7 g). Finally, the aqueous residue was extracted with 1 L BuOH and partitioned to obtain the BuOH extract (8.8 g).

An aliquot of the CH_2_Cl_2_ extract (3.0 g) was subsequently chromatographed on silica gel using a flash chromatographic gradient elution system from 100% (v/v) CH_2_Cl_2_ to 100% MeOH (v/v), to yield ten fractions (D1–D10). Fraction D3 (32 mg) was further purified on preparative normal-phase TLC (CH_2_Cl_2_/MeOH, 8:2 v/v) to furnish adociaquinone A **5** (7.0 mg). Fraction D4 (48.2 mg) was recrystallized in MeOH to obtain adociaquinone B **6** (18.5 mg). Fraction D5 (21 mg) was subjected to C6-phenyl reversed-phase HPLC using gradient ACN/H_2_O/HCOOH from 5/95/0.1 to 35/65/0.1 as eluent for 30 min (250 × 10 mm, flow rate: 3 mL/min, wavelength: 254 nm) to yield the mixture of xestoadociaminals C and D (**4a**, **4c**, 8.8 mg). Also, fraction D6 (47 mg) was subjected to C6-phenyl reversed-phase HPLC using gradient ACN/H_2_O/HCOOH from 5/95/0.1 to 35/65/0.1 as eluent for 30 min (flow rate: 3 mL/min, wavelength: 254 nm) to yield the mixture of xestoadociaminals A and B **3a**–**d** (15.0 mg) and 9.0 mg of the mixture of 15-chloro-14-hydroxyxestoquinone **9** and 14-chloro-15-hydroxyxestoquinone **10**. The mixture of **3a**–**d** was then recrystallized in MeOH to obtain mainly xestoadociaminal A **3a** (3.3 mg). Fractions D8 and D9 (69 mg) were further purified on Sephadex LH-20 column, eluted with MeOH to give secoadociaquinones A **7** (12.0 mg) and B **8** (5.0 mg). 

The EtOAc extract (1.7 g) was then chromatographed on silica gel using a flash chromatographic gradient elution system from 100% (v/v) CH_2_Cl_2_ to 100% MeOH (v/v), to give seven fractions (E1–E7). Fraction E3 (200 mg) was purified on a Sephadex LH-20 column (CH_2_Cl_2_/MeOH, 1:1 v/v) to give five sub-fractions (E3a–E3e). Fraction E3c was purified by preparative normal-phase TLC (CH_2_Cl_2_/MeOH, 8:2 v/v) to yield isomers of 15-chloro-14-hydroxyxestoquinone and 14-chloro-15-hydroxyxestoquinone **9** and **10** (2.5 mg). Fraction E4 (340 mg) was positive towards 2,2-diphenyl picrylhydrazyl DPPH antioxidant assay and was further purified on Sephadex LH-20 column (CH_2_Cl_2_/MeOH, 1:1 v/v) to give four sub-fractions (E4a–E4d). Sub-fraction E4a (73 mg) was purified by semi-preparative reversed-phase HPLC (Luna C-18 column 250 × 10 mm, ACN/H_2_O/HCOOH from 10/90/0.1 to 55/45/0.1, 30 min, 3 mL/min, 254 nm) to yield secoadociaquinones A and B **7** and **8** (5 mg). Fraction E4c (28 mg) was purified on preparative normal-phase TLC (CH_2_Cl_2_/MeOH, 8:2 v/v) to yield xestoquinol sulfate **11** (2 mg).

The butanol extract (2.7 g) was chromatographed on silica gel using a flash chromatographic gradient elution system from 100% (v/v) CH_2_Cl_2_ to 100% MeOH (v/v), to give seven fractions (B1–B7). Fraction B5 (CH_2_Cl_2_/MeOH, 8:2 v/v, 200 mg) was firstly purified on semi-preparative HPLC (Luna C-18 column, ACN/H_2_O/HCOOH 20/80/0.1 to 55/45/0.1, 3 mL/min, 254 nm) to yield three sub fractions B5a (60 mg), B5b (18 mg), and B5c (18 mg). Repeated purification of fraction B5b on semi-preparative HPLC (HILIC column, ACN/H_2_O/HCOOH 80/20/0.1, 3 mL/min, 254 nm) led to the isolation of xestoadociaquinones A and B **1a** and **1b** (1.7 mg) and xestoadociaminals A and B (**3a**, **3c**, 1.5 mg). Fraction B6 (CH_2_Cl_2_/MeOH, 1:1 v/v, 115 mg) was firstly purified on semi-preparative HPLC (HILIC column, ACN/H_2_O/HCOOH 80/20/0.1, 254 nm) to yield three sub-fractions B6a (20 mg), B6b (65 mg), and B6c (30 mg). After repeated purification of the sub fraction B6b on semi-preparative HPLC (HILIC column 250 × 10 mm, ACN/H_2_O/HCOOH 90/10/0.1, 3 mL/min, 254 nm) and preparative silica TLC (CH_2_Cl_2_/MeOH, 7:3 v/v), 14-carboxy-xestoquinol sulfate **2** (2.4 mg) was isolated.

Xestoadociaquinones A and B (**1a** and **1b**): pale yellow amorphous solid; ^1^H and ^13^C NMR spectral data, see Table 1; (+)-HRESIMS *m/z* 434.0905 [M + H]^+^ (calcd for C_20_H_20_NO_8_S, 434.0904).

14-Carboxy-xestoquinol sulfate (**2**): yellow amorphous solid, [α]_D_^25^ −17 (*c* 0.028, MeOH), UV (EtOH) λ_max_ (log ε): 207 (2.95) nm; IR (NaCl disk) ν_max_ 3318, 2924, 1740, 1458, 668 cm^−1^; ^1^H and ^13^C NMR spectral data, see Table 1; (−)-HRESIMS *m/z* 443.0447 [M−H]^+^ (calcd for C_21_H_15_O_9_S, 443.0442).

Xestoadociaminal A (**3a**): yellow amorphous solid, [α]_D_^25^ −55 (*c* 0.05, MeOH), UV (EtOH) λ_max_ (log ε): 205 (3.79), 231 (3.74), 251 (3.71), 314 (3.59) nm; IR (NaCl disk) ν_max_ 3649, 2924, 2855, 1752, 1717, 1508, 1458 cm^−1^; ^1^H and ^13^C NMR spectral data, see Table 1; (+)-HRESIMS *m/z* 468.1100 [M + H]^+^ (calcd for C_24_H_22_NO_7_S, 468.1111).

Xestoadociaminal B (**3c**): yellow amorphous solid; ^1^H and ^13^C NMR spectral data, see Table 1; (+)-HRESIMS *m/z* 468.1100 [M + H]^+^ (calcd for C_24_H_22_NO_7_S, 468.1111).

Xestoadociaminals C and D (**4a**, **4c**): yellow amorphous solid; ^1^H and ^13^C NMR spectral data, see Table 1; (+)-HRESIMS *m/z* 468.1100 [M + H]^+^ (calcd for C_24_H_22_NO_7_S, 468.1111).

### 3.4. Protein Kinase Assays 

Evaluation of the protein kinase inhibitory activity was performed *in vitro* as previously described [10]. Briefly, homogenization buffer: 60 mM β-glycerophosphate, 15 mM *p*-nitrophenylphosphate, 25 mM Mops (pH 7.2), 15 mM EGTA, 15 mM MgCl_2_, 1 mM dithiothreitol, 1 mM sodium vanadate, 1 mM NaF, 1 mM phenylphosphate, 10 µg leupeptin mL^−1^, 10 µg aprotinin mL^−1^, 10 µg soybean trypsin inhibitor mL^−1^, and 100 µg benzamidine. Buffer A: 10 mM MgCl_2_, 1 mM EGTA, 1 mM dithiothreitol, 25 mM Tris-HCl pH 7.5, 50 µg heparin mL^−1^. Buffer C: homogenization buffer but 5 mM EGTA, no NaF and no protease inhibitors. Kinase activities were assayed in triplicates in buffer A or C at 30 °C, at a final ATP concentration of 15 µM. The order of mixing the reagents was: buffers, substrate, enzyme, inhibitor and ^33^P-radiolabelled ATP. Isolated compounds were tested against a panel of eight kinases; namely cyclin-dependent kinase 1 (CDK1/cyclin B), cyclin-dependent kinase 2 (CDK2/cyclin A), cyclin-dependent kinase 5 (CDK5/p25), cyclin-dependent kinase 9 (CDK9/cyclin T), casein kinase 1 (CK1), Cdc2-like kinase 1 (CLK1), dual-specificity tyrosine-(Y)-phosphorylation regulated kinase 1A (DYRK1A) and glycogen synthase kinase-3α/β (GSK-3α/β).

### 3.5. Antibacterial Assay

The antibacterial activity assay was conducted on the bacterial strain Gram-positive *Staphylococcus aureus* ATCC 6538 and Gram-negative *Escherichia coli* ATCC 8739 evaluated *in vitro* by determining the IC_50_. A pre-culture of 5 mL LB (Luria Bertoni) medium was prepared by inoculating a colony of the bacterial strain and was incubated at 37 °C with stirring overnight. The concentration of the pre-culture was assessed by measuring the optical density OD at 620 nm and adjusted by dilution to obtain a suspension of 0.03 OD. The IC_90_ was determined by a liquid test in 96-well-plates. A quantity of 200 µL of the bacterial suspension was distributed in each well and 10 µL of the extracts, fractions or pure compounds solutions in DMSO (10, 5 and 2 mg/mL, respectively) were added in triplicate. The 96 well-plates were incubated at 30 °C for 16 to 18 h with shaking (450 rpm). The optical density of the wells was measured at 620 nm and the results were interpreted by calculating the percentage of growth inhibition in each well using the formula: % inhibition = 100 − (DOS − DOB)/(DOT − DOB) ×100 where T = bacterial suspension without test sample, B = culture medium without bacteria and S = bacterial suspension test sample. Ampicillin and chloramphenicol were used as positive control against *S. aureus* and *E. coli*, respectively.

### 3.6. Antioxidant Assay: Qualitative DPPH Screening

The potential antioxidant activity of sponge crude extract and fractions from different chromatography procedures was evaluated using the scavenging activity of the DPPH (2,2-diphenyl-1-picrylhydrazyl) free radicals. Active fractions were visualized by spraying a purple DPPH solution (2 mg/mL in MeOH) on a TLC, where extracts or fractions have been deposited. Immediate discoloration of DPPH around tested samples reveals their antioxidant activity. The well-known antioxidant ascorbic acid was used as positive control.

## Supplementary Information

Structures of new compounds, 1D and 2D NMR spectra, and MS spectra of the new compounds (**1a**, **1b**, **2**, **3a**, **3c**, **4a**, **4c**).
