# Supplementary material for: Novel Adociaquinone Derivatives from the Indonesian Sponge Xestospongia sp
Source: Mar Drugs. 2015 Apr 28;13(5):2617–28. doi: 10.3390/md13052617 (PMC4446597; doi:10.3390/md13052617)
Supplement: Supplementary file 1 [file marinedrugs-13-02617-s001.pdf]

Supplementary Information

S1. General Experimental Procedures
Figure S1. ^{1}H NMR spectrum of Xestoadociaquinone A (**1a**) and B (**1b**) in CD_3OD (600 MHz)
Figure S2. DEPT NMR spectrum of Xestoadociaquinone A (**1a**) and B (**1b**) in CD_3OD (150 MHz)
Figure S3. ^{1}H-^{1}H COSY spectrum of Xestoadociaquinone A (**1a**) and B (**1b**) in CD_3OD (600 MHz)
Figure S4. HSQC spectrum of Xestoadociaquinone A (**1a**) and B (**1b**) in CD_3OD (600 MHz)
Figure S5. HMBC spectrum of Xestoadociaquinone A (**1a**) and B (**1b**) in CD_3OD (600 MHz)
Figure S6. NOESY spectrum of Xestoadociaquinone A (**1a**) and B (**1b**) in CD_3OD (600 MHz)
Figure S7. ^{1}H NMR spectrum of 14-carboxy-xestoquinol sulfate (**2**) in DMSO-d_6 (600 MHz)
Figure S8. ^{1}H-^{1}H COSY spectrum of 14-carboxy-xestoquinol sulfate (**2**) in DMSO-d_6 (600 MHz)
Figure S9. DEPT spectrum of 14-carboxy-xestoquinol sulfate (**2**) in DMSO-d_6 (150 MHz)
Figure S10. HSQC spectrum of 14-carboxy-xestoquinol sulfate (**2**) in DMSO-d_6 (600 MHz)
Figure S11. HMBC spectrum of 14-carboxy-xestoquinol sulfate (**2**) in DMSO-d_6 (600 MHz)
Figure S12. NOESY spectrum of 14-carboxy-xestoquinol sulfate (**2**) in DMSO-d_6 (600 MHz)
Figure S13. ^{1}H NMR spectrum of Xestoadociaminal A (**3a**) in DMSO-d_6 (600 MHz)
Figure S14. DEPT NMR spectrum of Xestoadociaminal A (**3a**) in DMSO-d_6 (150 MHz)
Figure S15. ^{1}H-^{1}H COSY spectrum of Xestoadociaminal A (**3a**) in DMSO-d_6 (600 MHz)
Figure S16. HSQC spectrum of Xestoadociaminal A (**3a**) in DMSO-d_6 (600 MHz)
Figure S17. HMBC spectrum of Xestoadociaminal A (**3a**) in DMSO-d_6 (600 MHz)
Figure S18. NOESY spectrum of Xestoadociaminal A (**3a**) in DMSO-d_6 (600 MHz)
Figure S19. ^{1}H NMR spectrum of the mixture of Xestoadociaminals A (**3a**) and B (**3c**) in DMSO-d_6 (600 MHz)
Figure S20. DEPT NMR spectrum of the mixture of Xestoadociaminals A (**3a**) and B (**3c**) in DMSO-d_6 (150 MHz)22
Figure S21. ^{1}H-^{1}H COSY spectrum of the mixture of Xestoadociaminals A (**3a**) and B (**3c**) in DMSO-d_6 (600 MHz)
Figure S22. HSQC spectrum of the mixture of Xestoadociaminals A (**3a**) and B (**3c**) in DMSO-d_6 (600 MHz)
Figure S23. HMBC spectrum of the mixture of Xestoadociaminals A (**3a**) and B (**3c**) in DMSO-d_6 (600 MHz)

Figure S24. NOESY spectrum of the mixture of Xestoadociaminals A (**3a**) and B (**3c**) in DMSO-*d*6 (600 MHz)
Figure S25. ^{1}H NMR spectrum of the mixture of Xestoadociaminals C (**4a**) and D (**4c**) in DMSO-d_6 (600 MHz)
Figure S26. DEPT spectrum of the mixture of Xestoadociaminals C (**4a**) and D (**4c**) in DMSO-d_6 (150 MHz)
Figure S27. ^{1}H-^{1}H COSY spectrum of the mixture of Xestoadociaminals C (**4a**) and D (**4c**) in DMSO-d_6 (600 MHz)
Figure S28. HSQC spectrum of the mixture of Xestoadociaminals C (**4a**) and D (**4c**) in DMSO-d_6 (600 MHz)
Figure S29. HMBC spectrum of the mixture of Xestoadociaminals C (**4a**) and D (**4c**) in DMSO-d_6 (600 MHz)
Figure S30. NOESY spectrum of the mixture of Xestoadociaminals C (**4a**) and D (**4c**) in DMSO-d_6 (600 MHz)

S1. General Experimental Procedures

Optical rotations were recorded on a Perkin-Elmer 341 polarimeter (Villebon-sur-Yvette, France). IR spectra were recorded on a FT-IR Shimadzu 8400 S spectrometer (Noisiel, France). UV spectra were recorded on a UVIKON 930 spectrometer (Kontron, France). Mass spectra were recorded on an API Q-STAR PULSAR I of Applied Biosystems (Concord, ON, Canada). NMR spectra were obtained on either a Bruker Avance 400 or 600 spectrometer (Wissenburg, France) using standard pulse sequences. The acquisition of HMBC spectra was optimized for either 7 or 8.3 Hz. Column chromatography (CC) purifications were performed using silica gel (200~400 mesh; Merck, Darmstadt, Germany) and Sephadex LH-20 (Amersham Pharmacia, Uppsala, Sweden). Fractions were analyzed by TLC using aluminum-backed sheets (Silica gel 60 F254) and visualized under UV (254 nm) and Lieberman spray reagent. Preparative TLC used glass plate coated with silica gel 60 F254, 0.25 mm thick (Merck, Darmstadt, Germany). Flash chromatography was carried out on Buchi C-615 pump system (Rungis, France). Analytical and semi-preparative reverse-phase (Gemini C6-phenyl, Luna RP18 and HILIC, Phenomenex, Le Pecq, France) columns were performed with an Alliance HPLC apparatus (model 2695, Waters, Saint-Quentin en Yvelines, France), equipped with a photodiode array detector (model 2998, Waters), an evaporative light-scattering detector (model Sedex 80, Sedere, Alforville, France), and the Empower software.

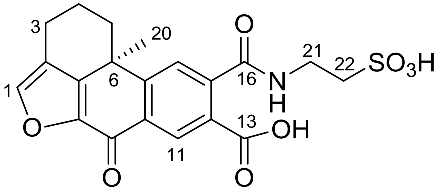

1a: Xestoadociaquinone A

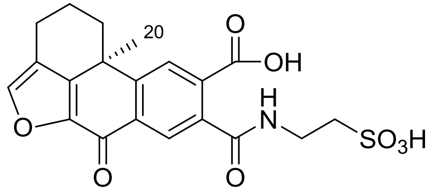

1b: Xestoadociaquinone B

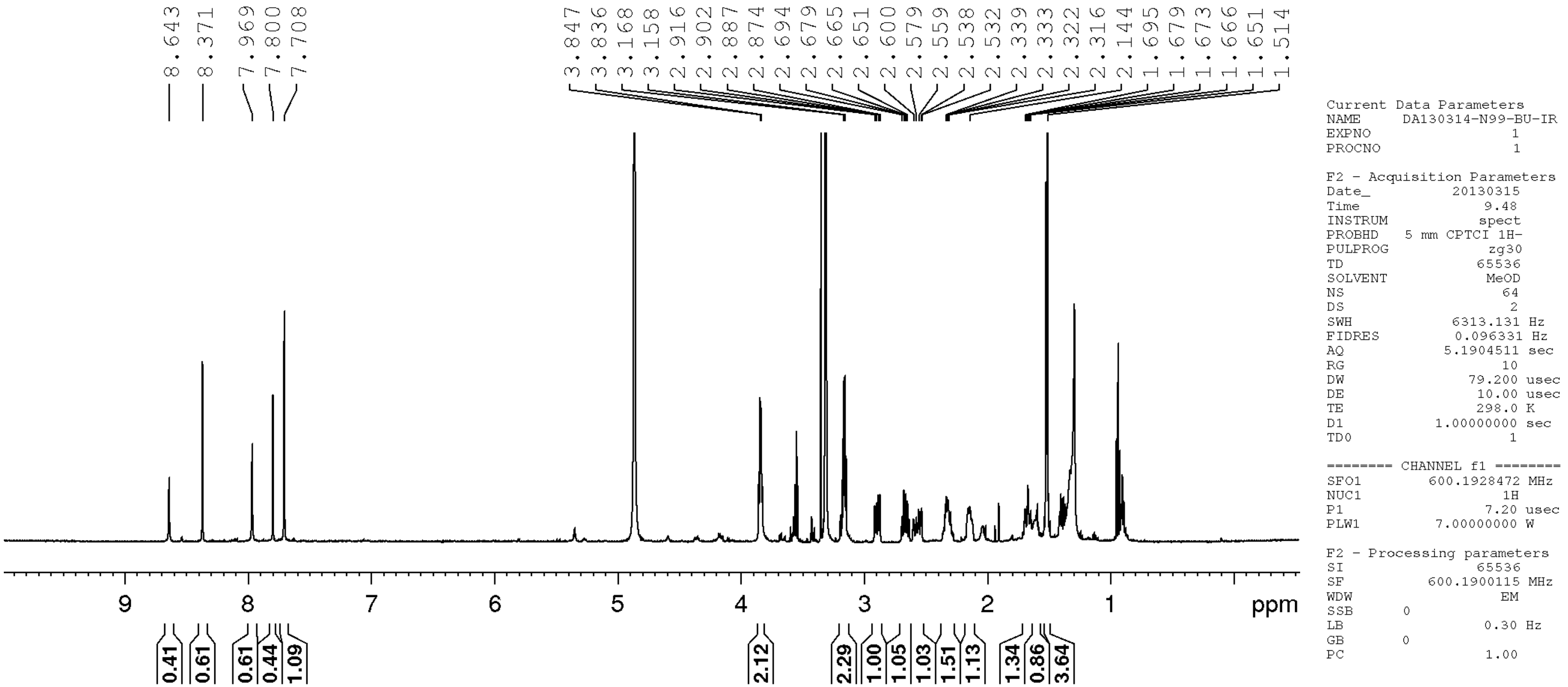

Figure S1. ^{1}H NMR spectrum of Xestoadociaquinone A (1a) and B (1b) in CD_3OD (600 MHz).

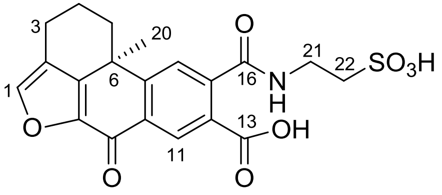

1a: Xestoadociaquinone A

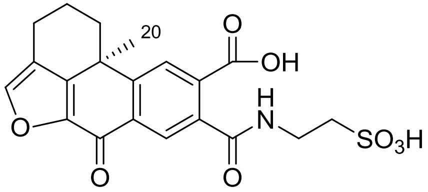

1b: Xestoadociaquinone B

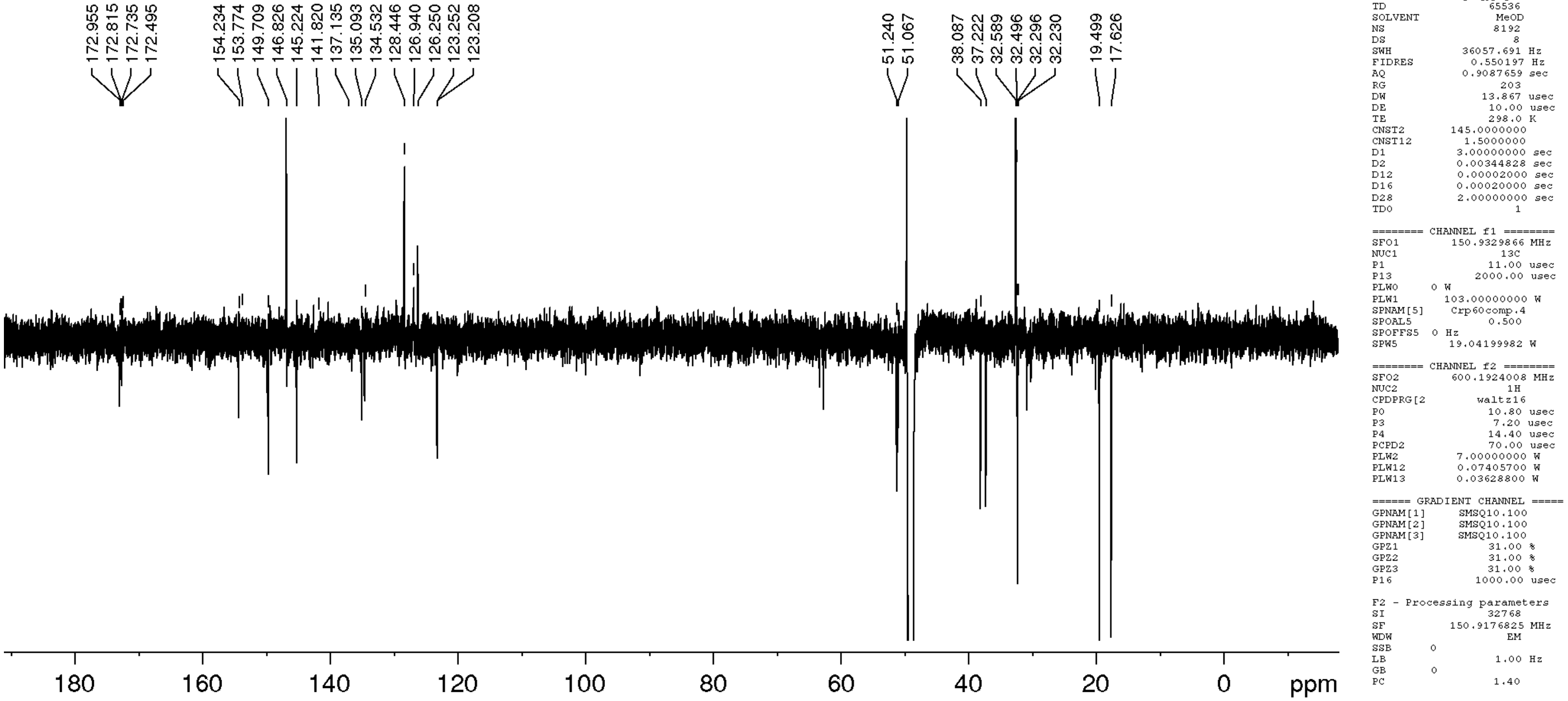

Figure S2. DEPT NMR spectrum of Xestoadociaquinone A (1a) and B (1b) in CD_3OD (150 MHz).

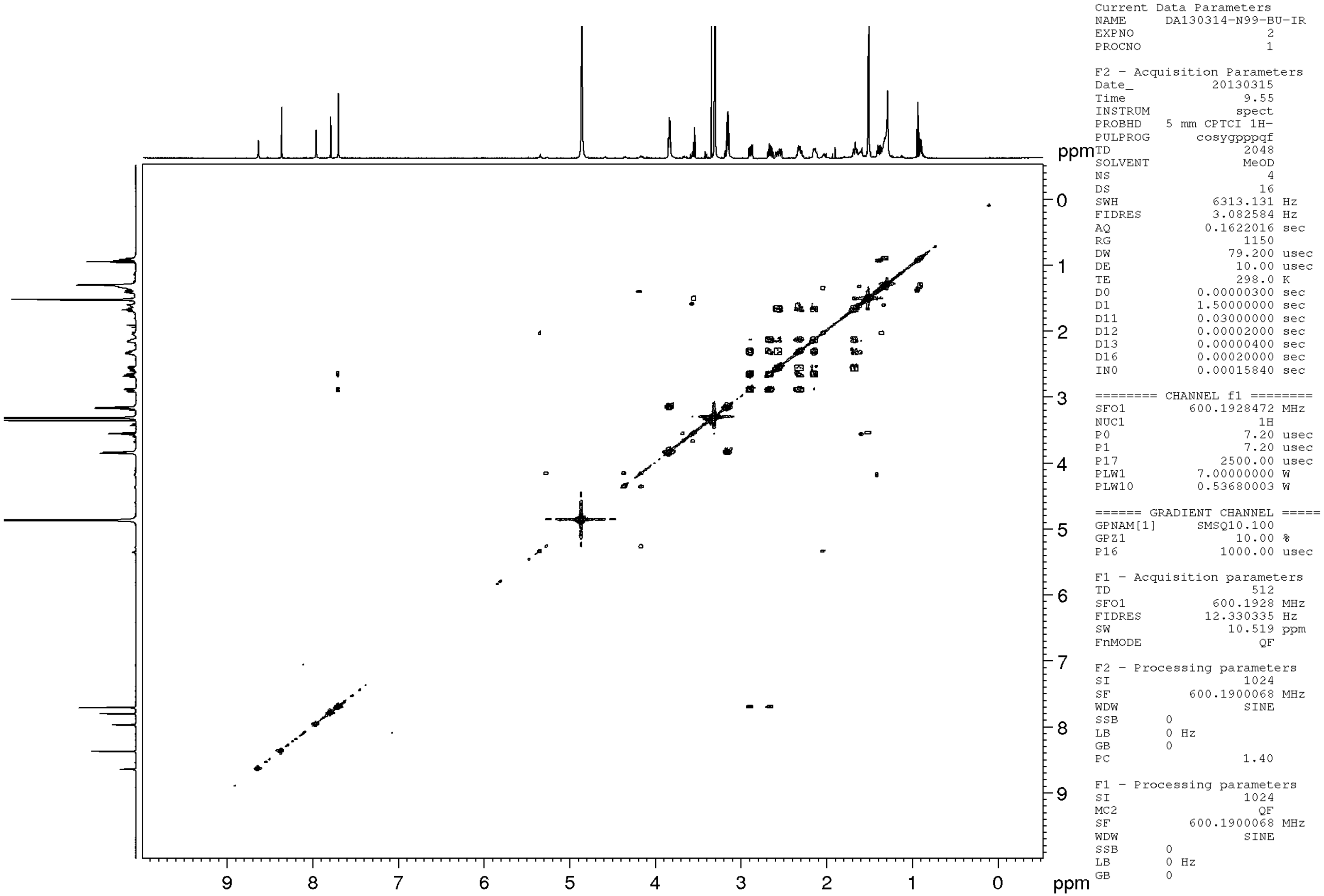

Figure S3. ^{1}H-^{1}H COSY spectrum of Xestoadociaquinone A (1a) and B (1b) in CD_3OD (600 MHz).

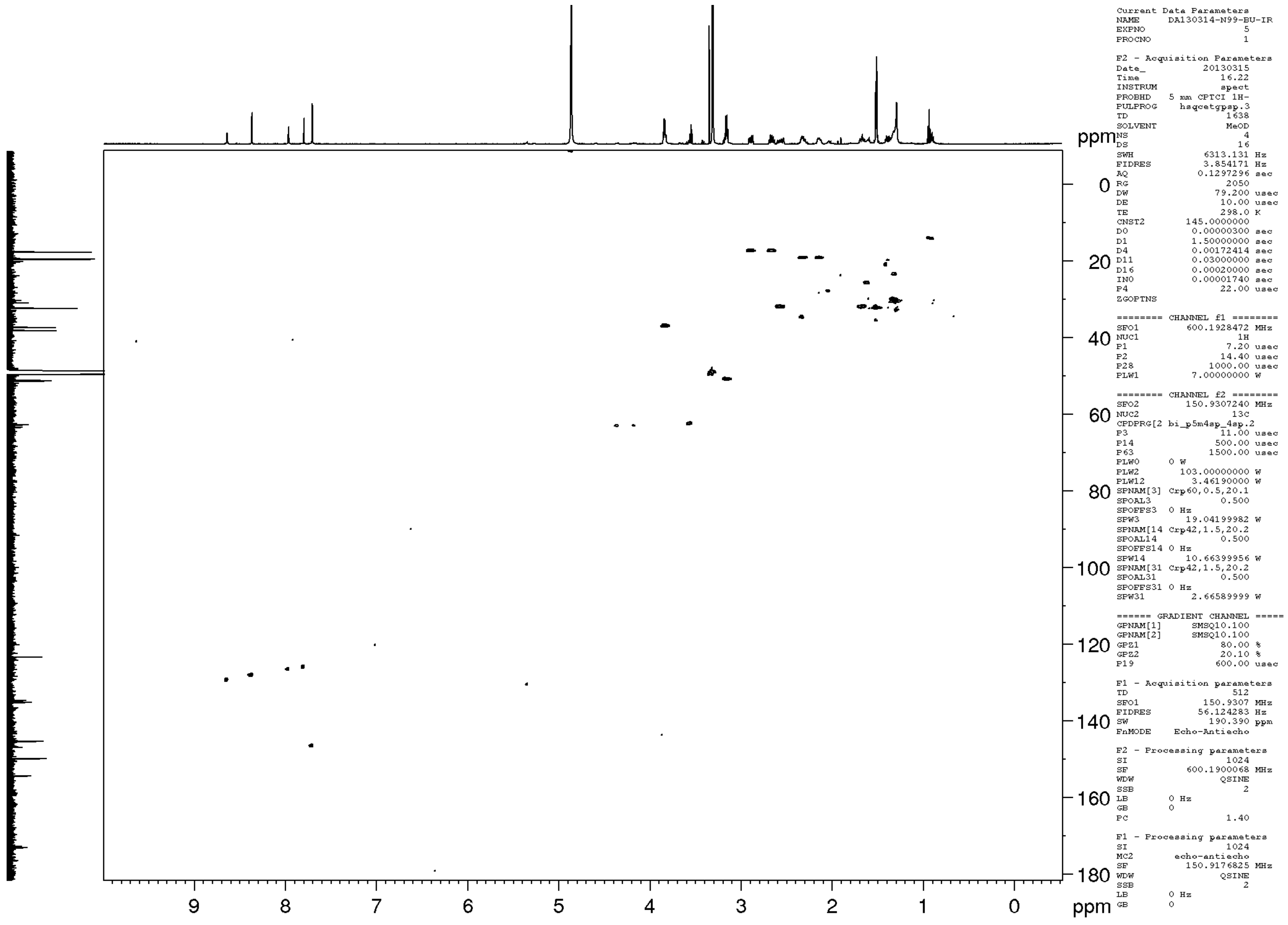

Figure S4. HSQC spectrum of Xestoadociaquinone A (1a) and B (1b) in CD_3OD (600 MHz).

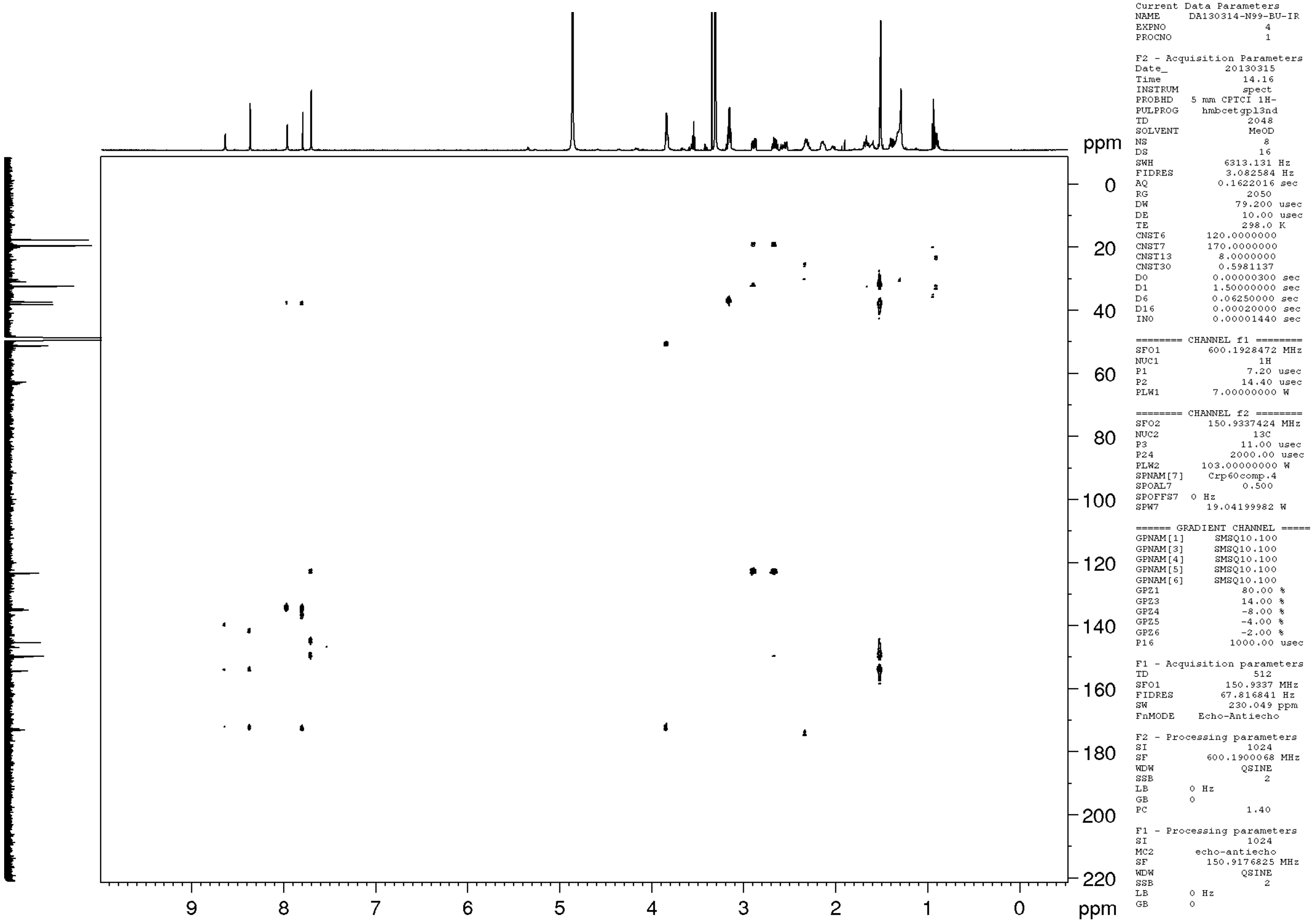

Figure S5. HMBC spectrum of Xestoadociaquinone A (1a) and B (1b) in CD_3OD (600 MHz).

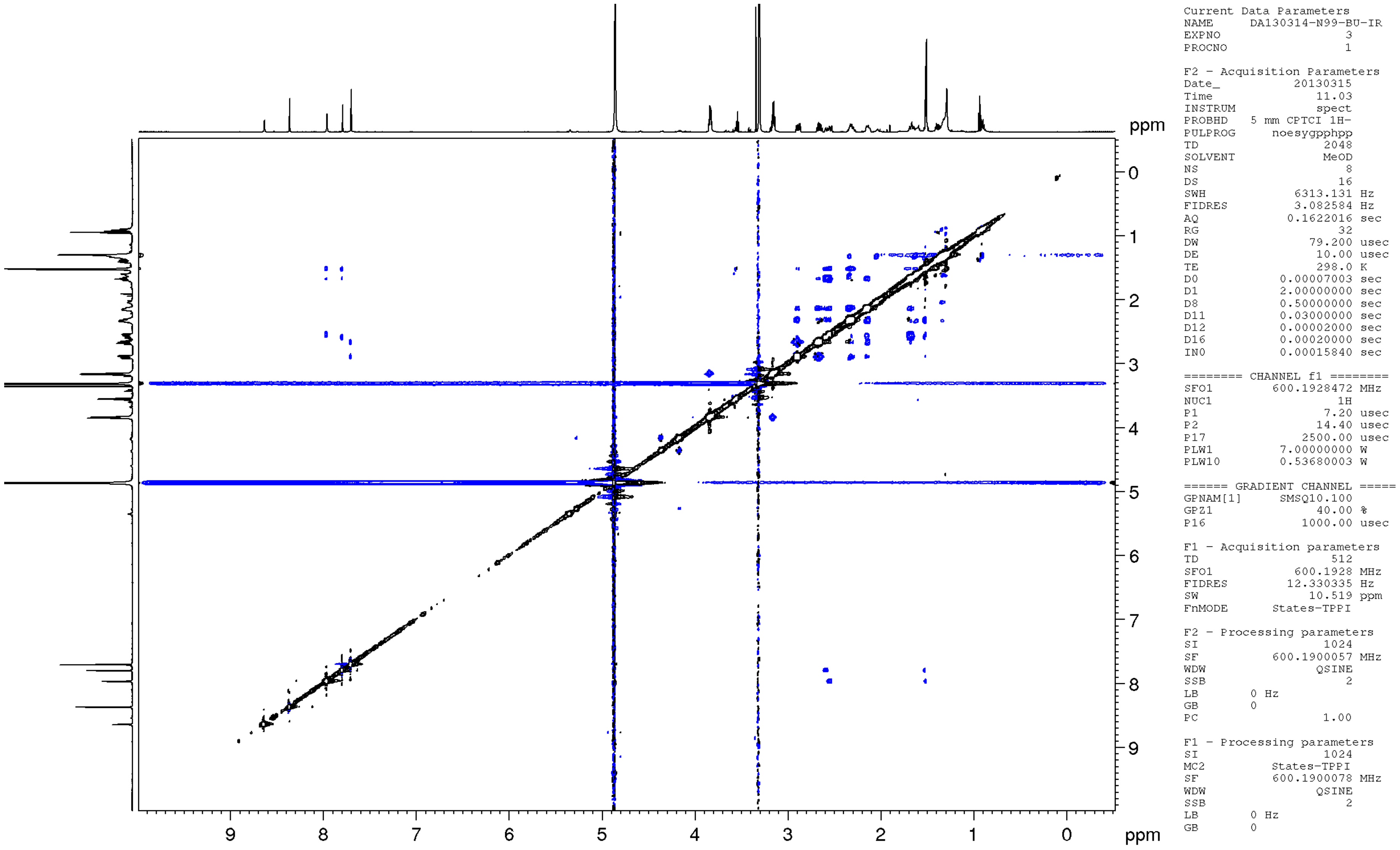

Figure S6. NOESY spectrum of Xestoadociaquinone A (1a) and B (1b) in CD_3OD (600 MHz).

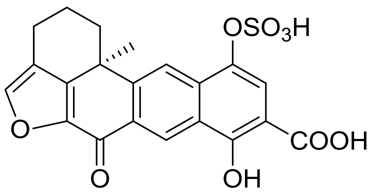

2: 14-carboxy-xestoquinol sulfate

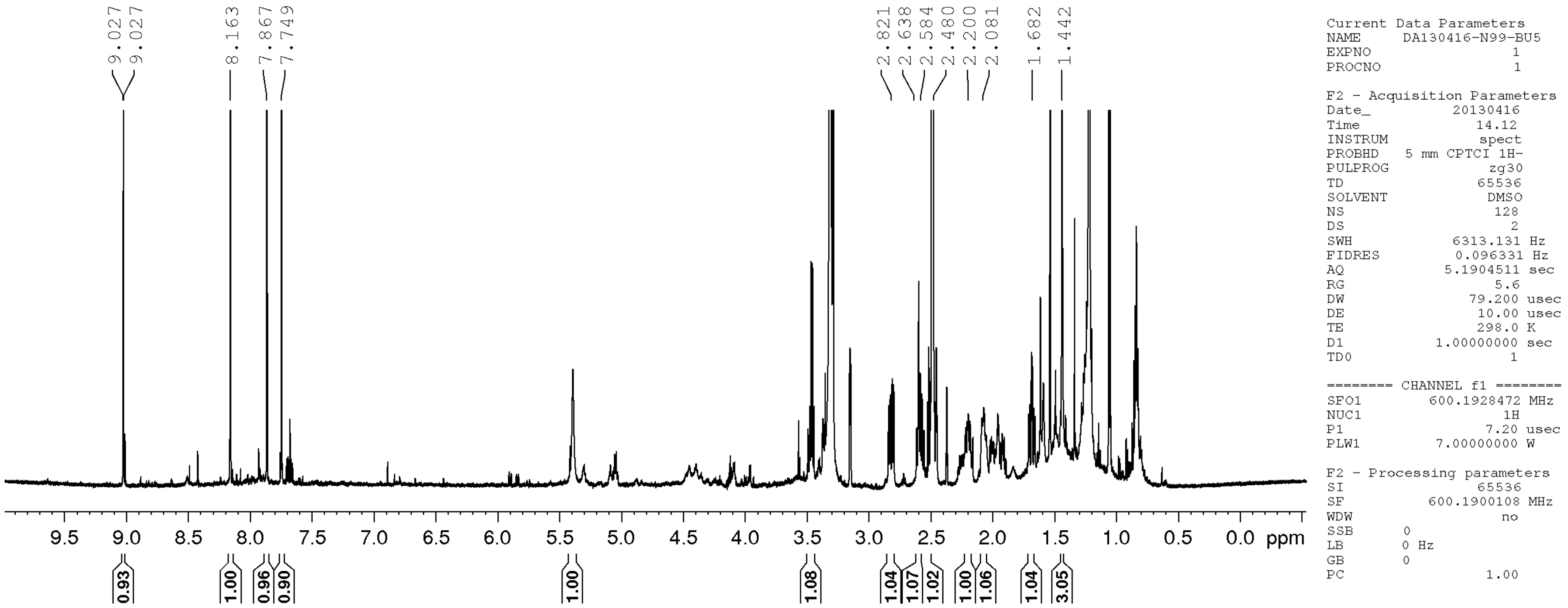

Figure S7. ^{1}H NMR spectrum of 14-carboxy-xestoquinol sulfate (2) in DMSO-d_6 (600 MHz).

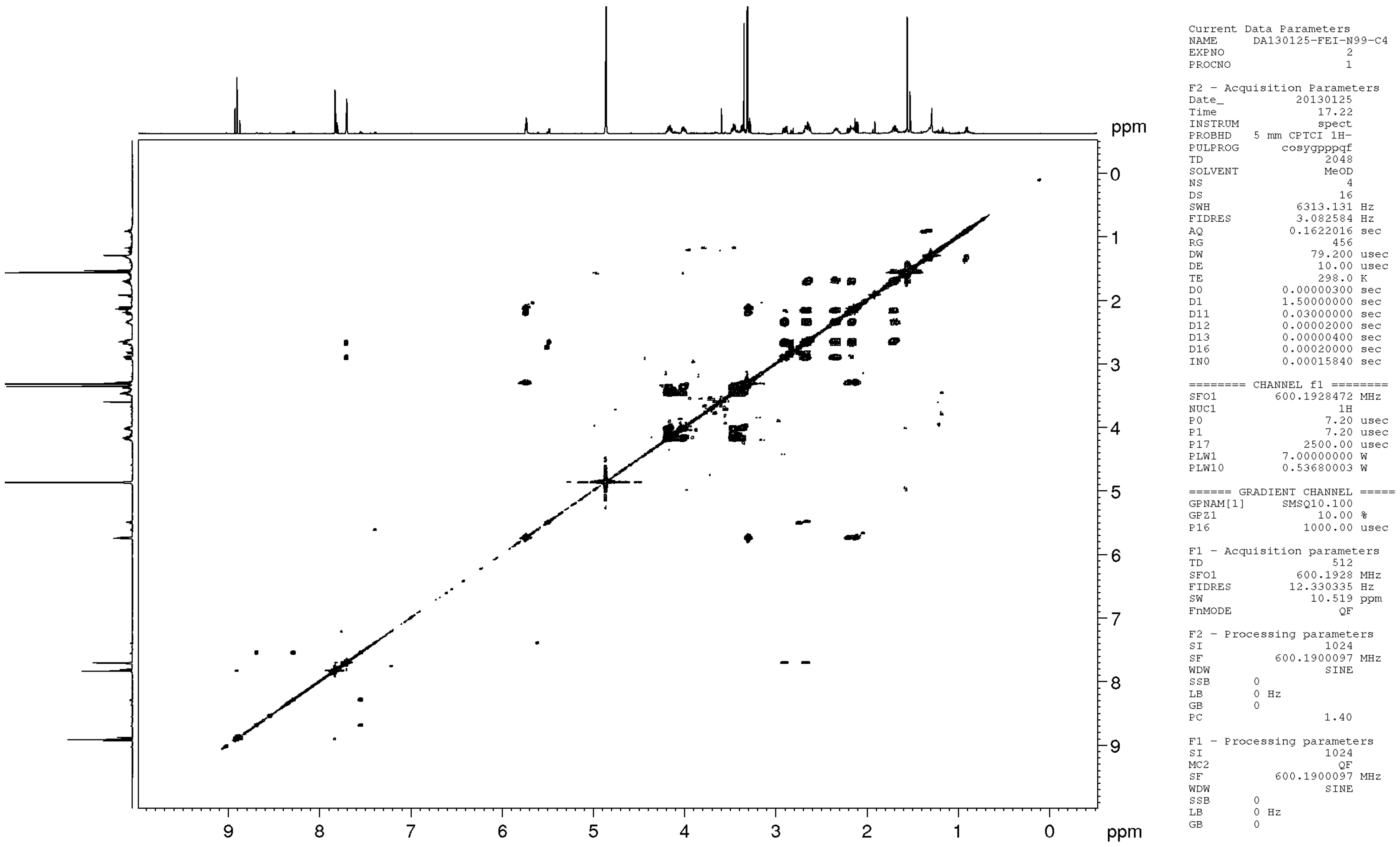

Figure S8. ^{1}H-^{1}H COSY spectrum of 14-carboxy-xestoquinol sulfate (2) in DMSO-d_6 (600 MHz).

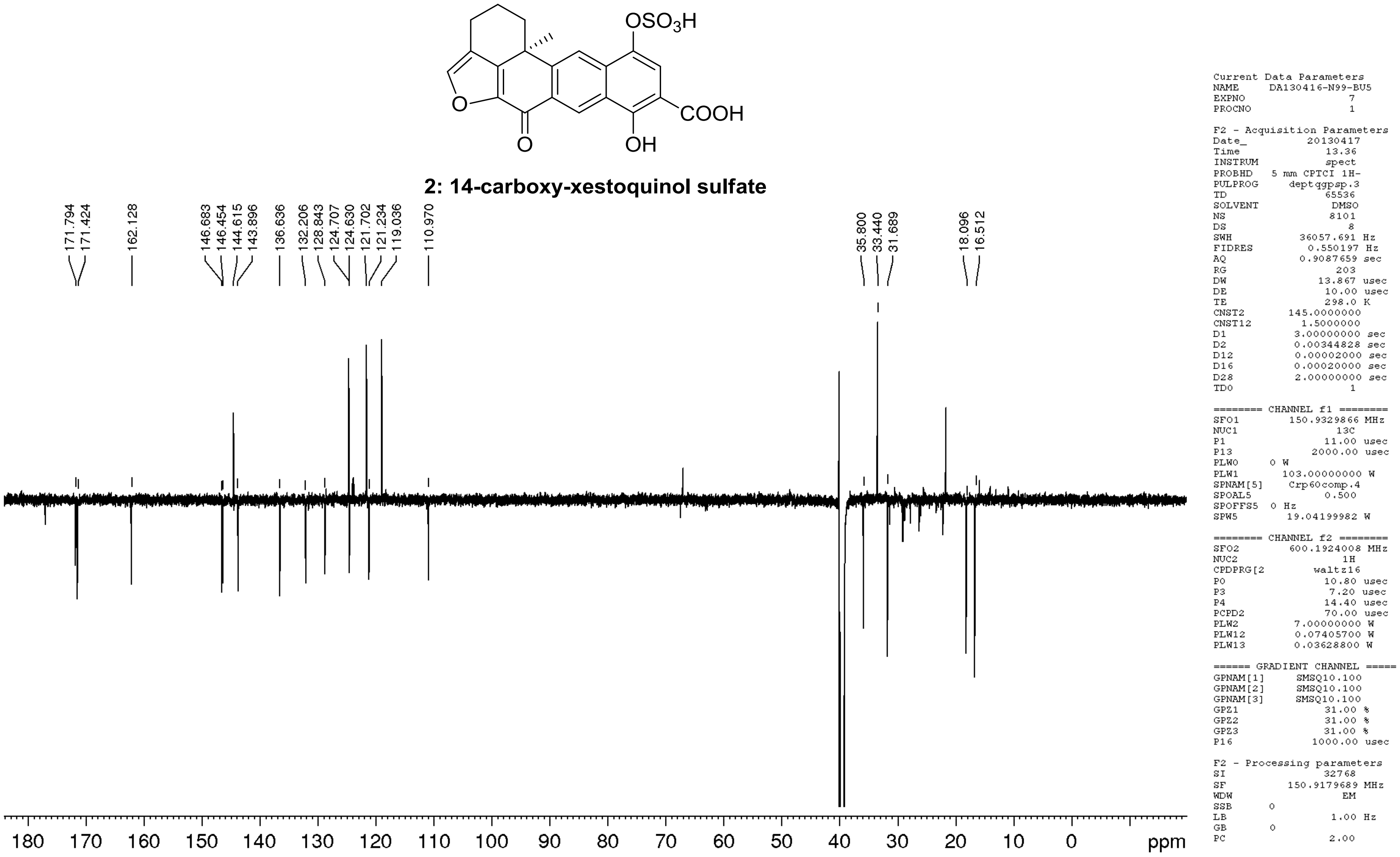

Figure S9. DEPT spectrum of 14-carboxy-xestoquinol sulfate (2) in DMSO-d_6 (150 MHz).

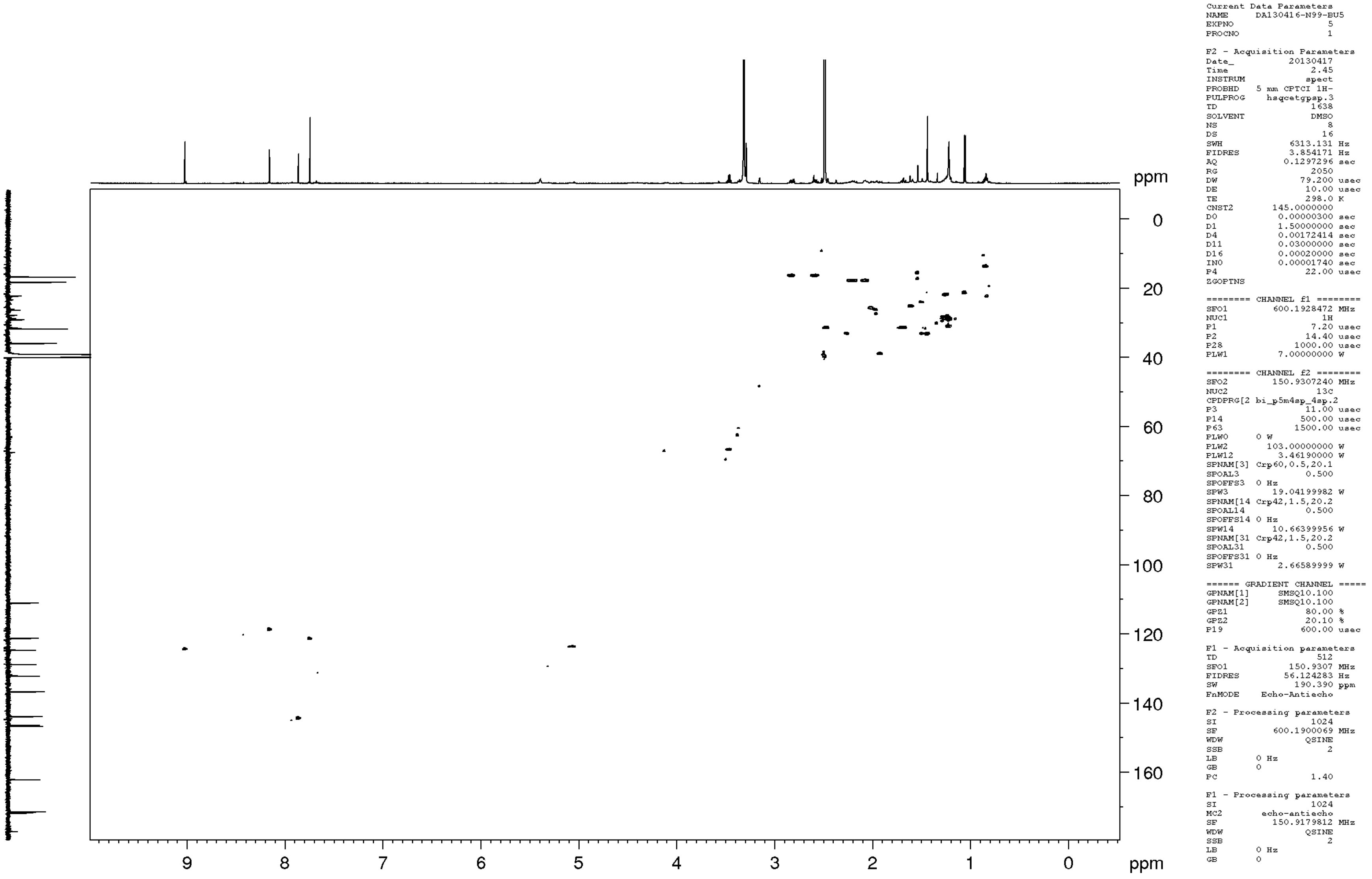

Figure S10. HSQC spectrum of 14-carboxy-xestoquinol sulfate (2) in DMSO-d_6 (600 MHz).

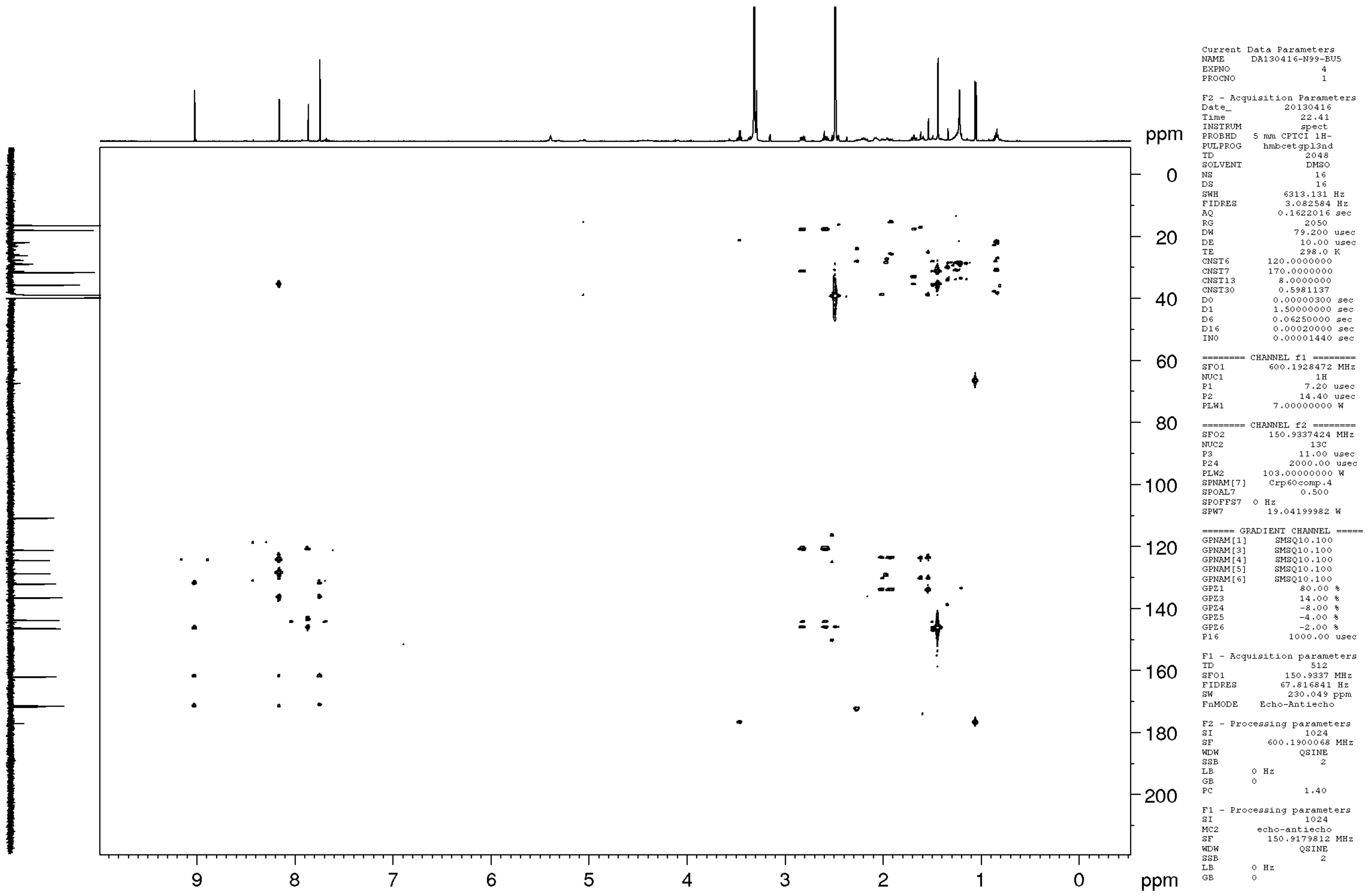

Figure 11. HMBC spectrum of 14-carboxy-xestoquinol sulfate (2) in DMSO-d_6 (600 MHz).

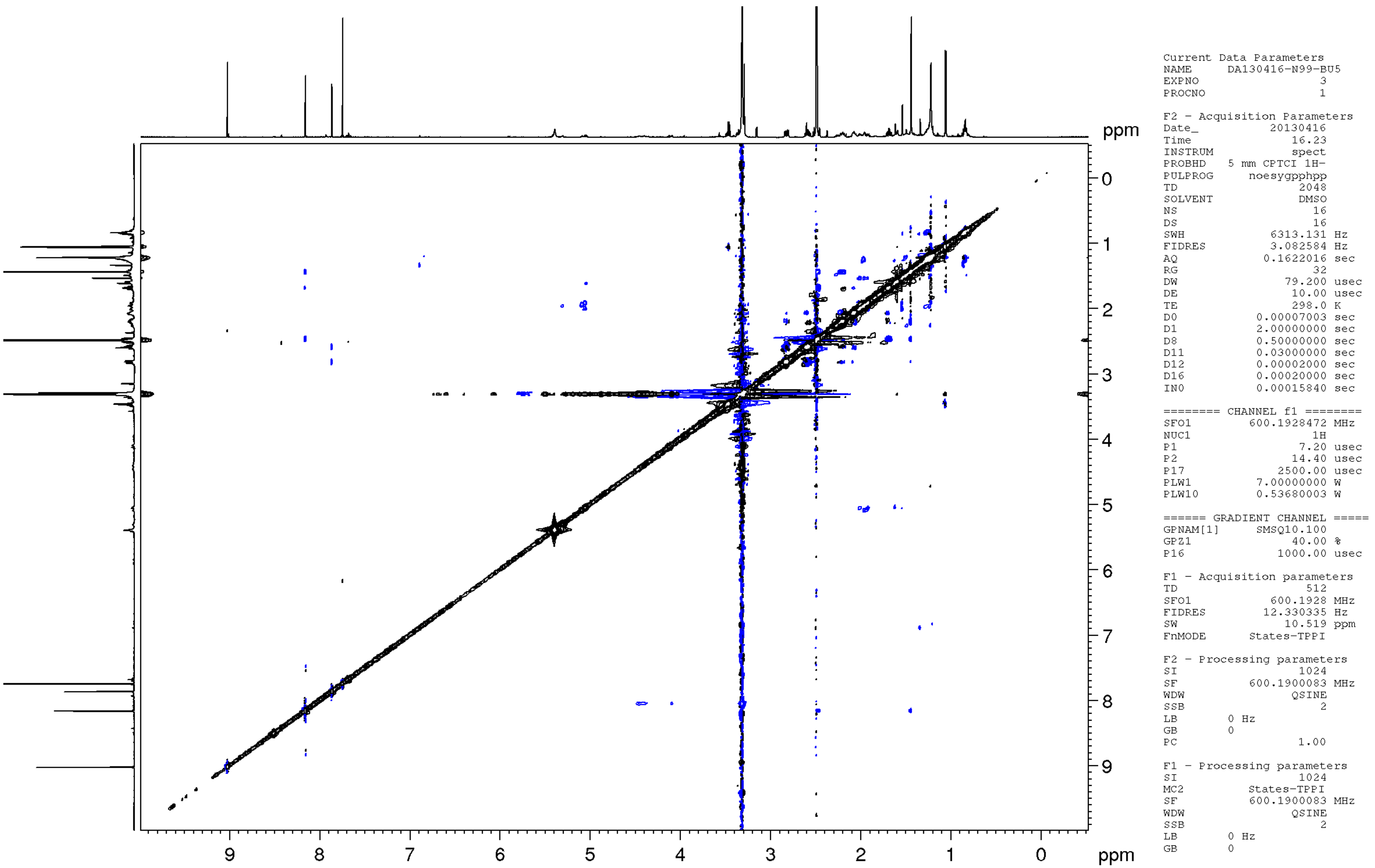

Figure S12. NOESY spectrum of 14-carboxy-xestoquinol sulfate (2) in DMSO-d_6 (600 MHz).

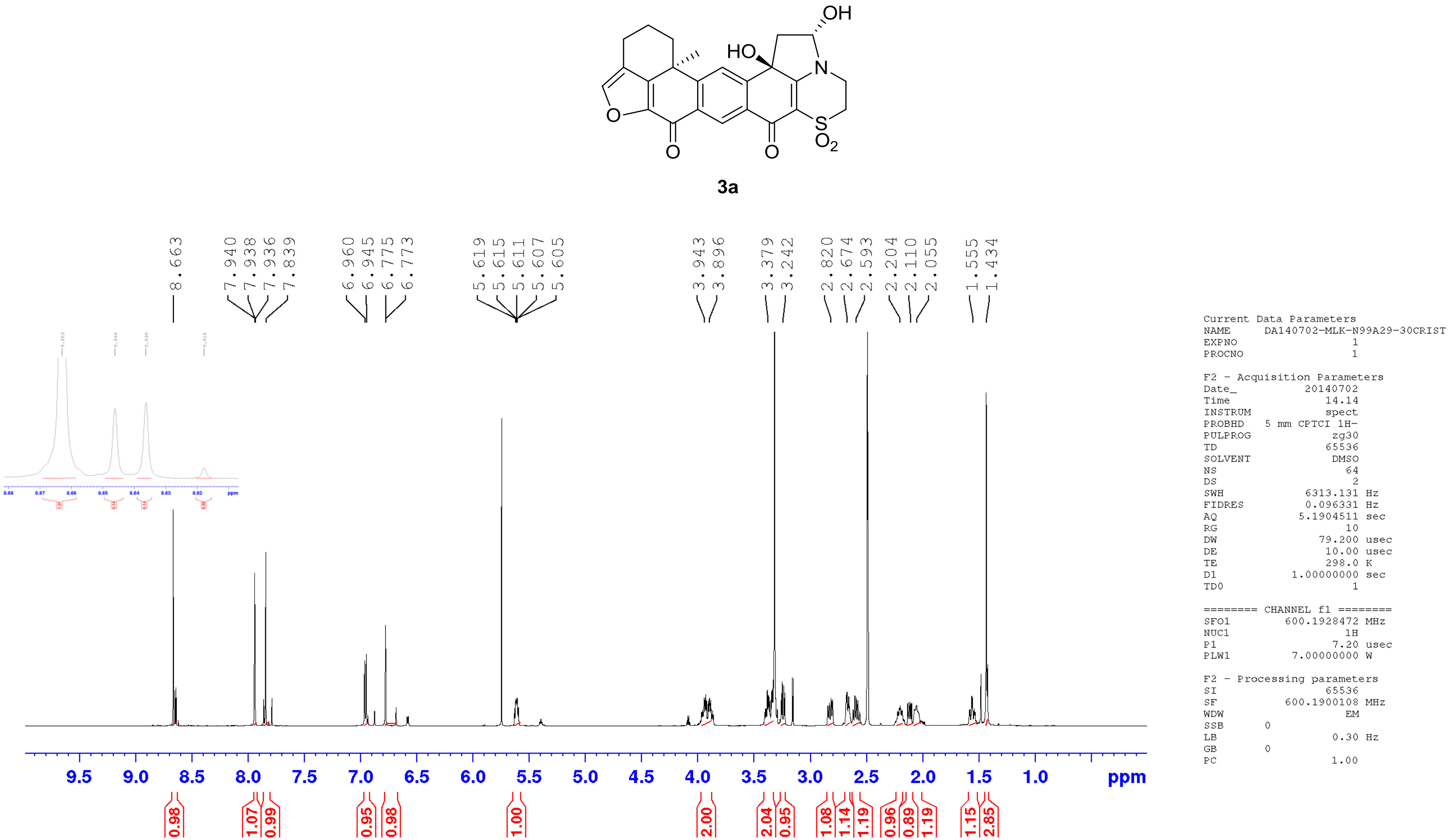

Figure S13. ^{1}H NMR spectrum of Xestoadociaminal A (3a) in DMSO-d_6 (150 MHz).

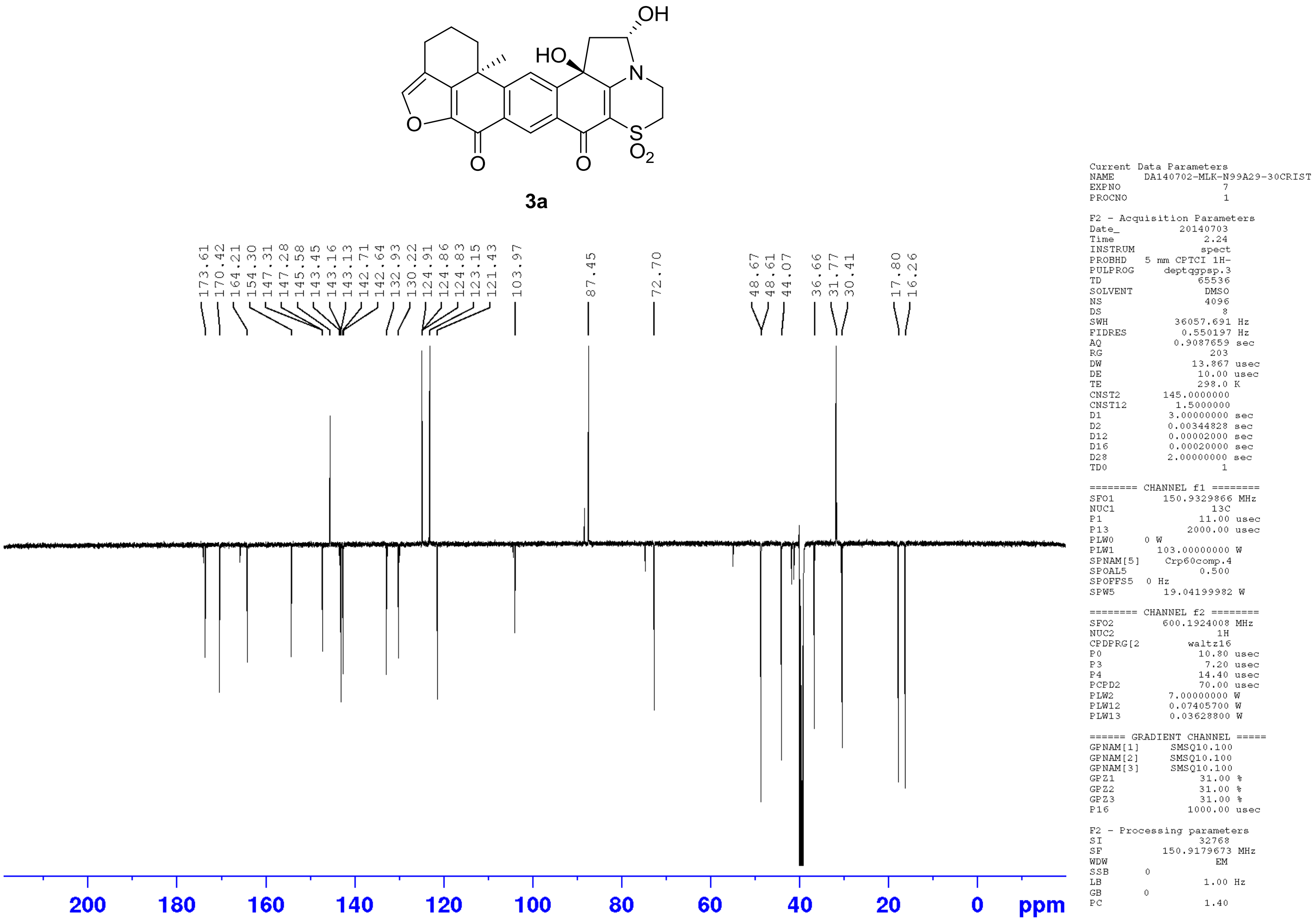

Figure S14. DEPT NMR spectrum of Xestoadociaminal A (3a) in DMSO-d_6 (150 MHz).

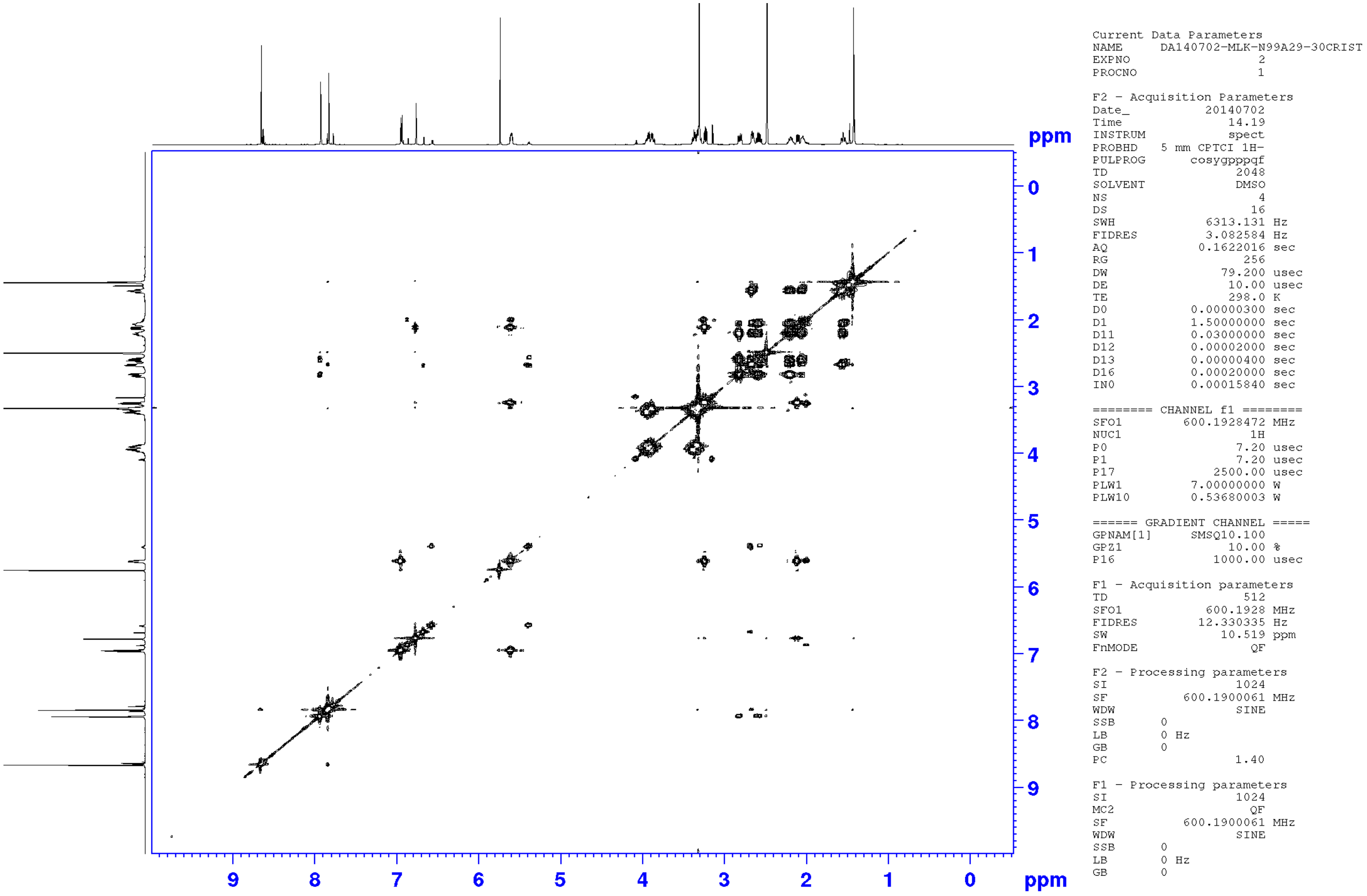

Figure S15. ^{1}H-^{1}H COSY spectrum of Xestoadociaminal A (3a) in DMSO-d_6 (600 MHz).

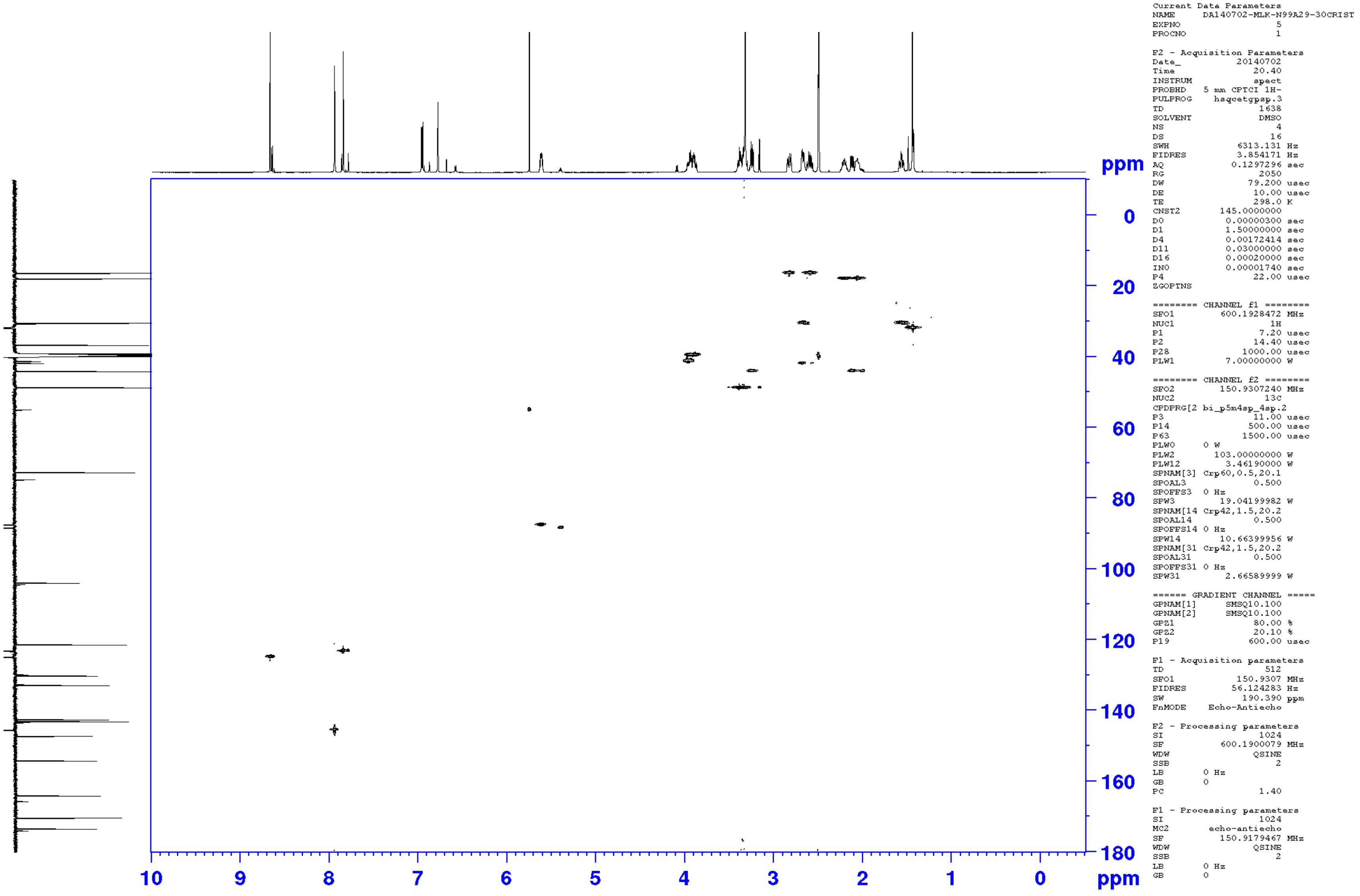

Figure S16. HSQC spectrum of Xestoadociaminal A (3a) in DMSO-d_6 (600 MHz).

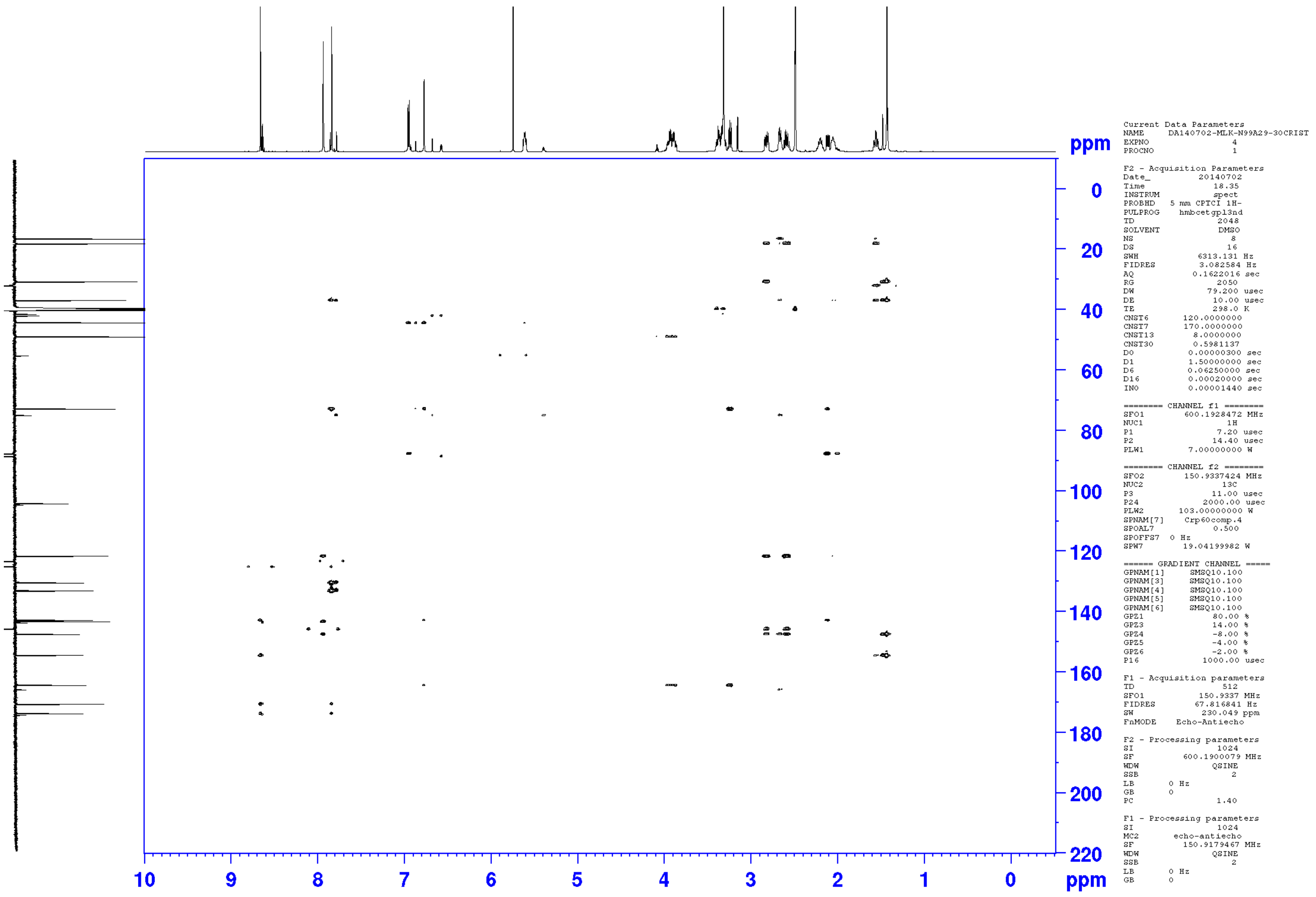

Figure S17. HMBC spectrum of Xestoadociaminal A (3a) in DMSO-d_6 (600 MHz).

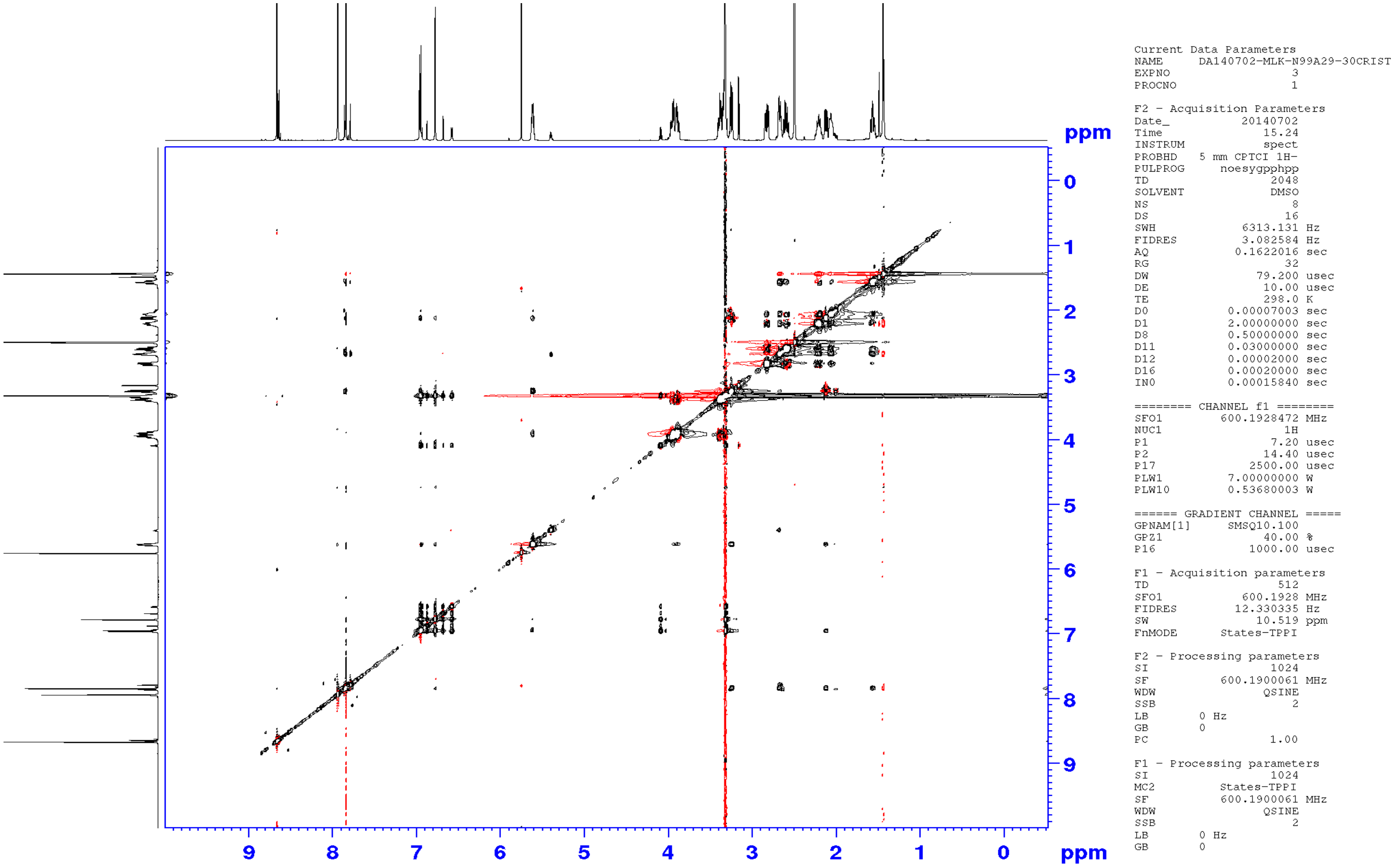

Figure S18. NOESY spectrum of Xestoadociaminal A (3a) in DMSO-d_6 (600 MHz).

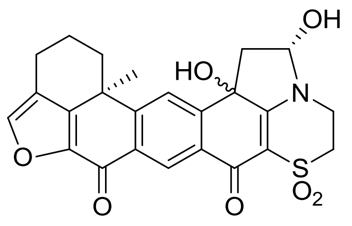

3a + 3c

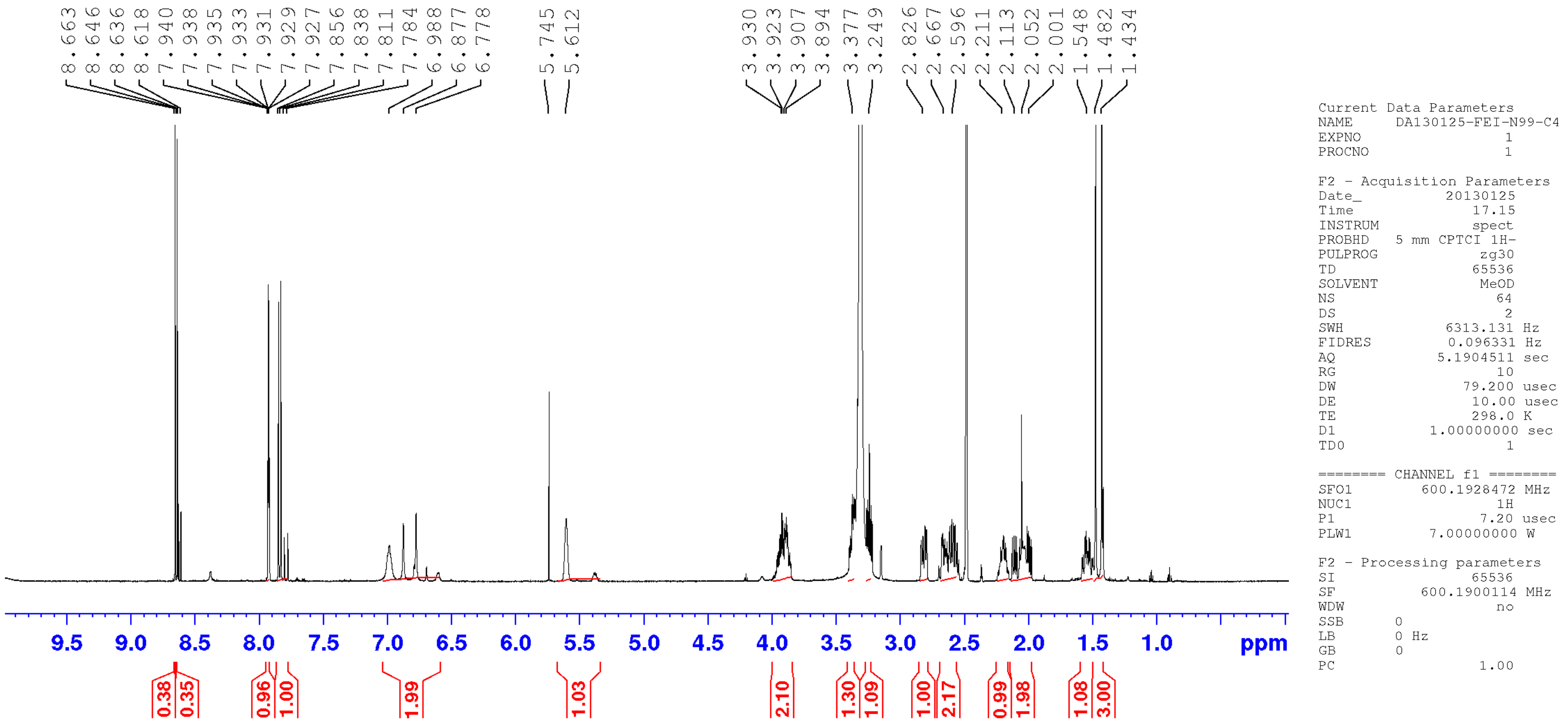

Figure S19. ^{1}H NMR spectrum of the mixture of Xestoadociaminals A (3a) and B (3c) in DMSO-d_6 (600 MHz).

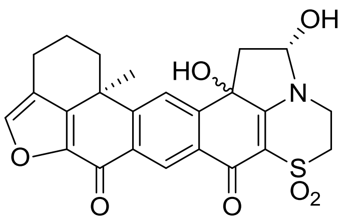

3a + 3c

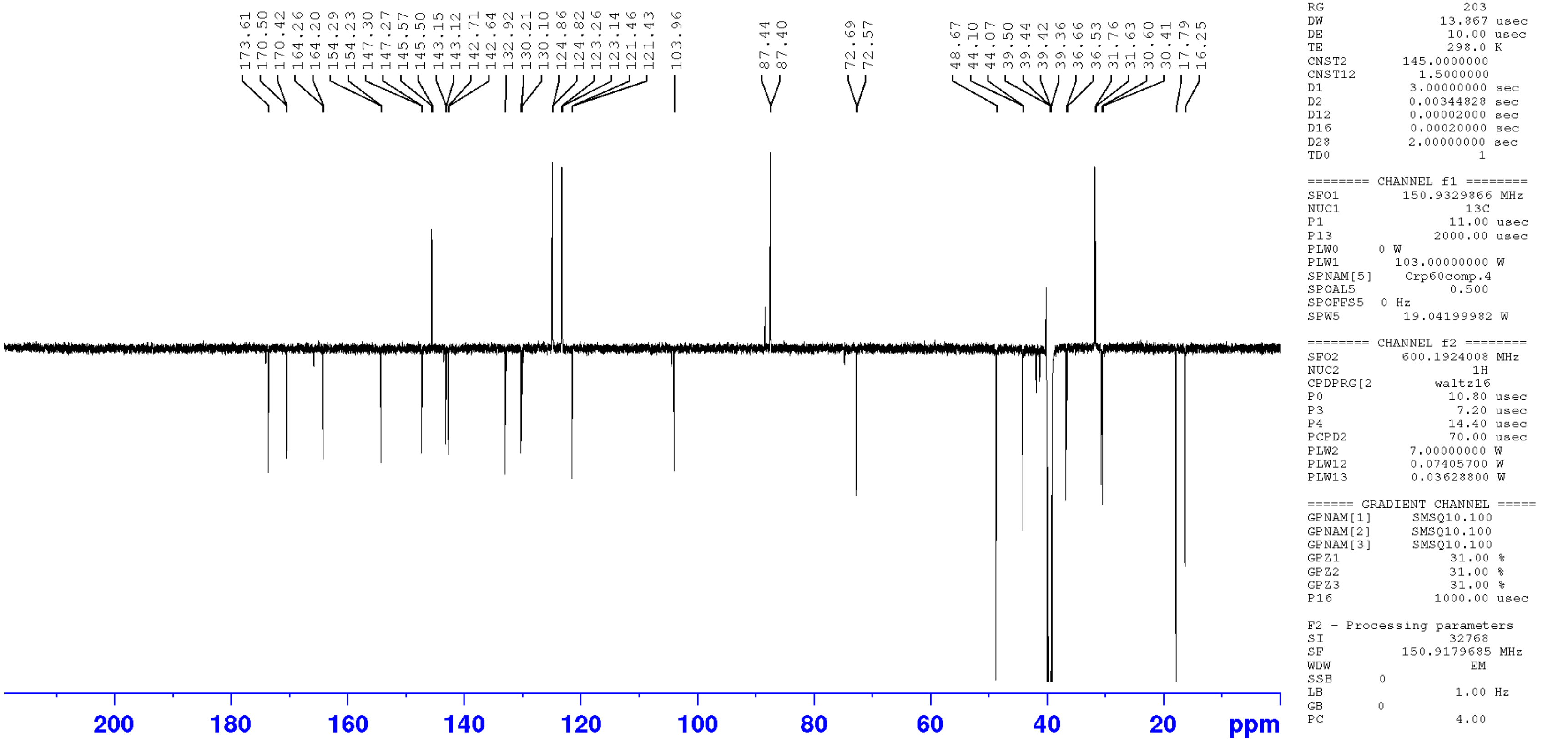

Figure S20. DEPT NMR spectrum of the mixture of Xestoadociaminals A (3a) and B (3c) in DMSO-d_6 (150 MHz).

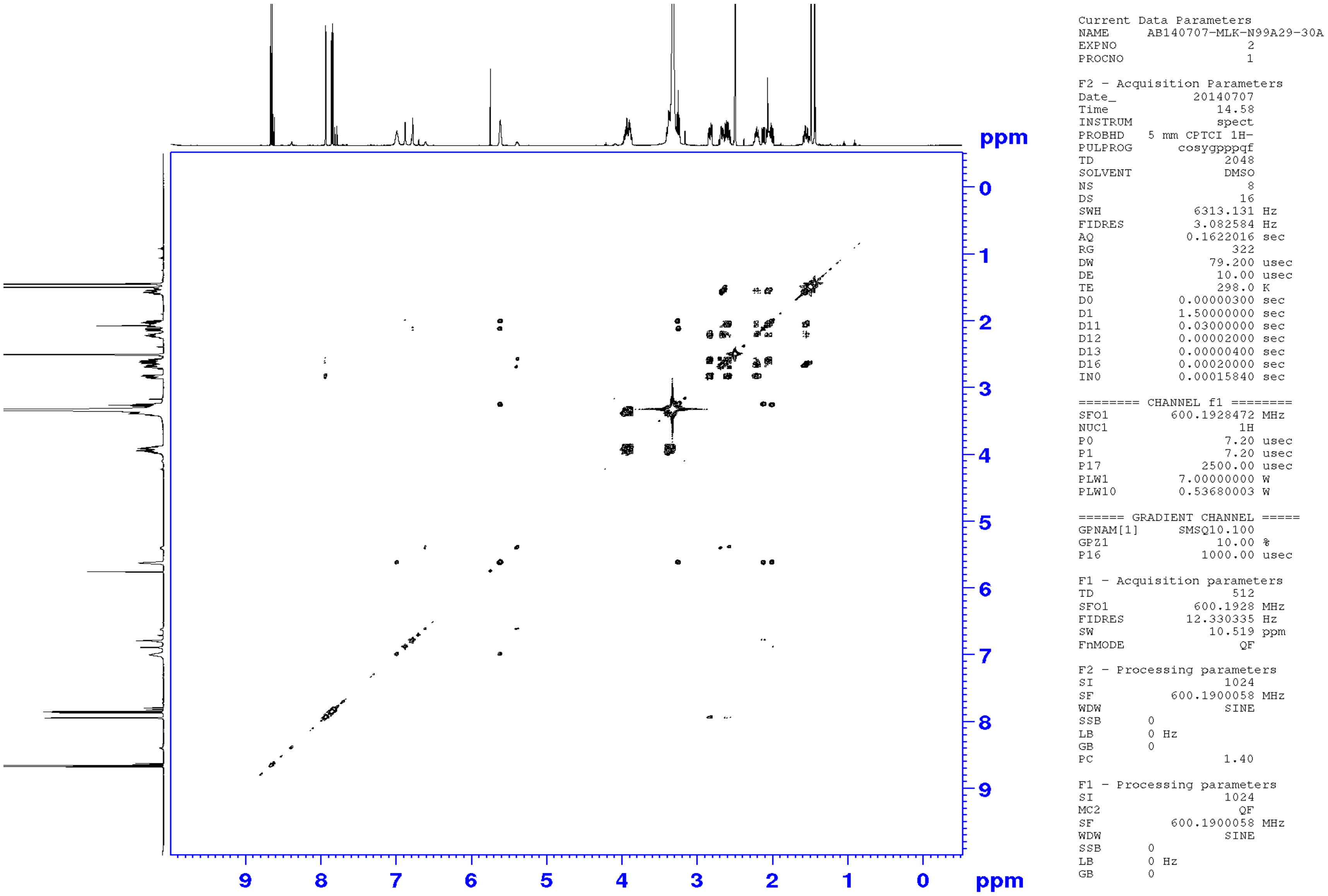

Figure S21. ^{1}H-^{1}H COSY spectrum of the mixture of Xestoadociamінals A (3a) and B (3c) in DMSO-d_6 (600 MHz).

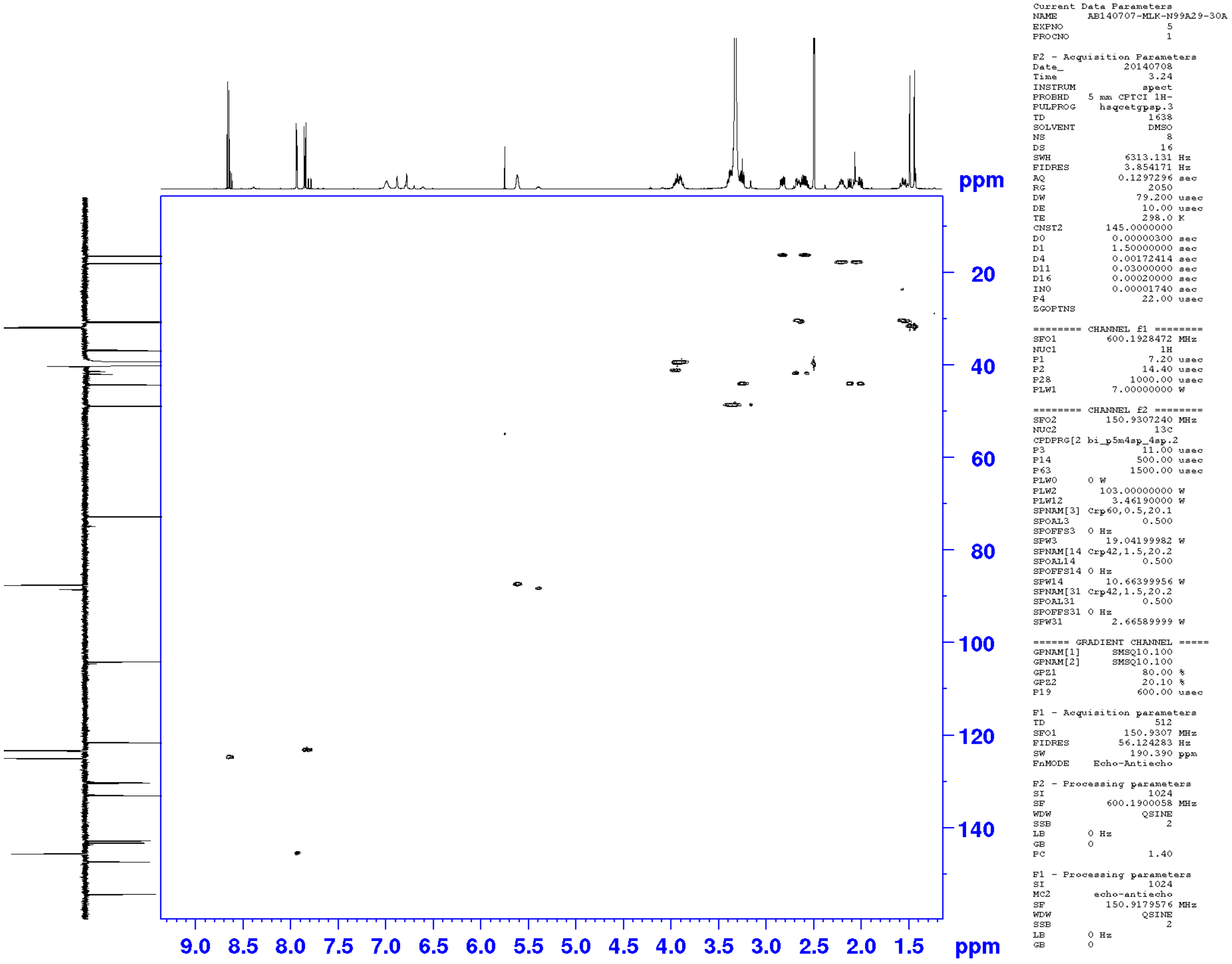

Figure S22. HSQC spectrum of the mixture of Xestoadociaminals A (3a) and B (3c) in DMSO-d_6 (600 MHz).

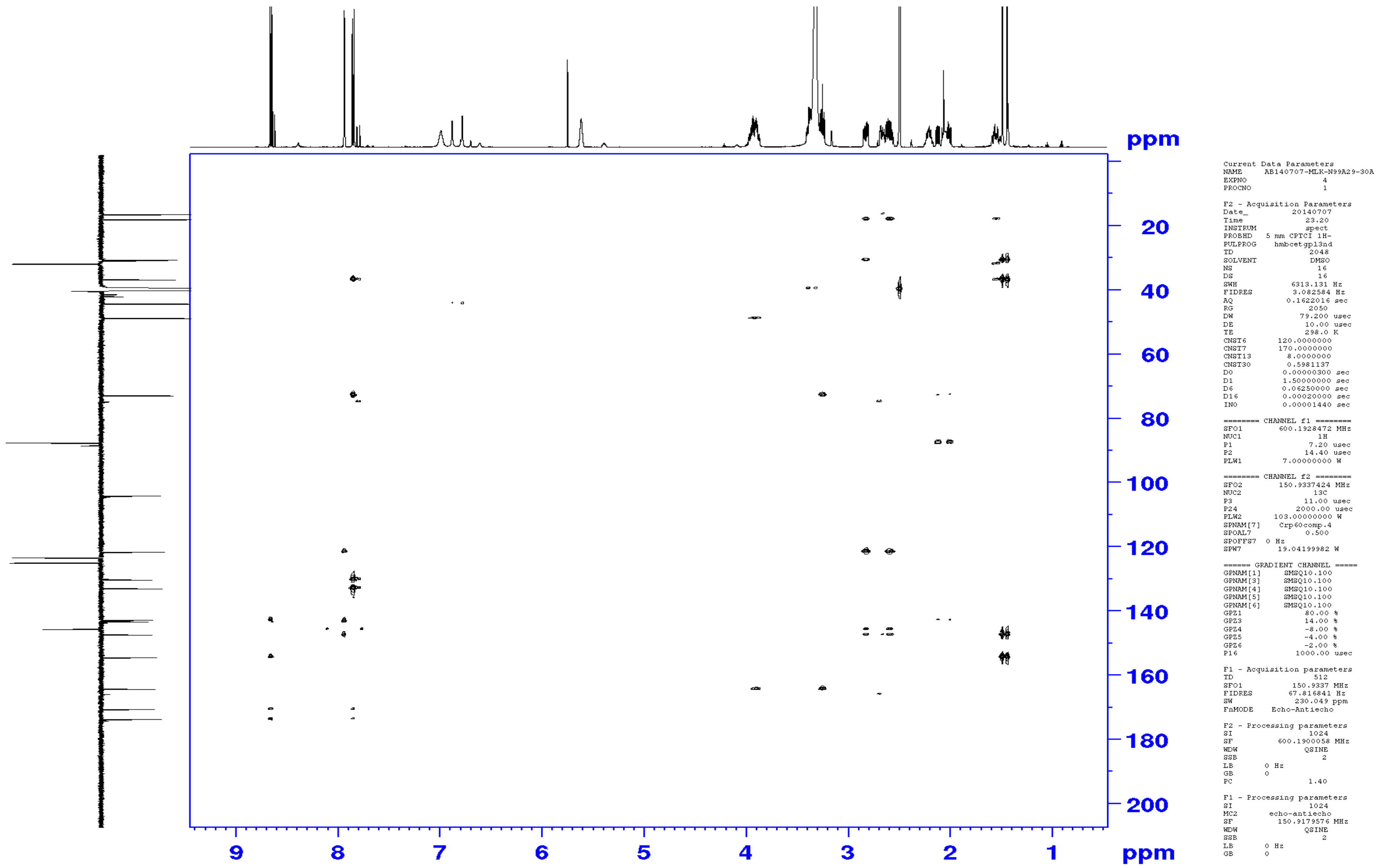

Figure S23. HMBC spectrum of the mixture of Xestoadociaminals A (3a) and B (3c) in DMSO-d_6 (600 MHz).

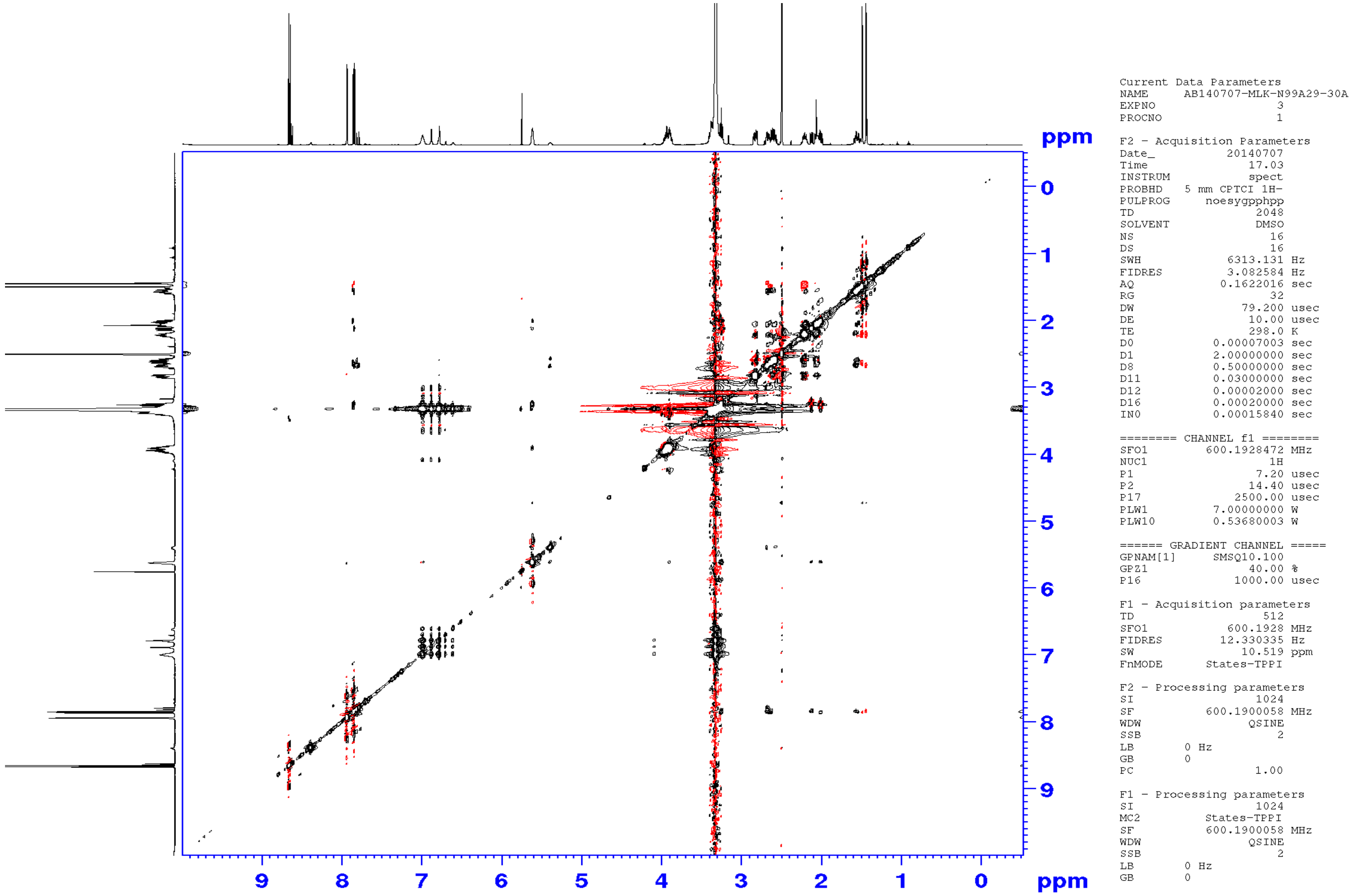

Figure S24. NOESY spectrum of the mixture of Xestoadociaminals A (3a) and B (3c) in DMSO-d_6 (600 MHz).

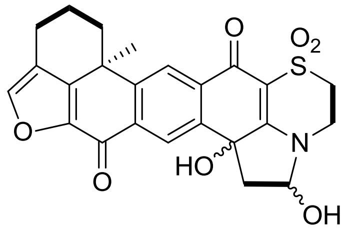

4a+4c

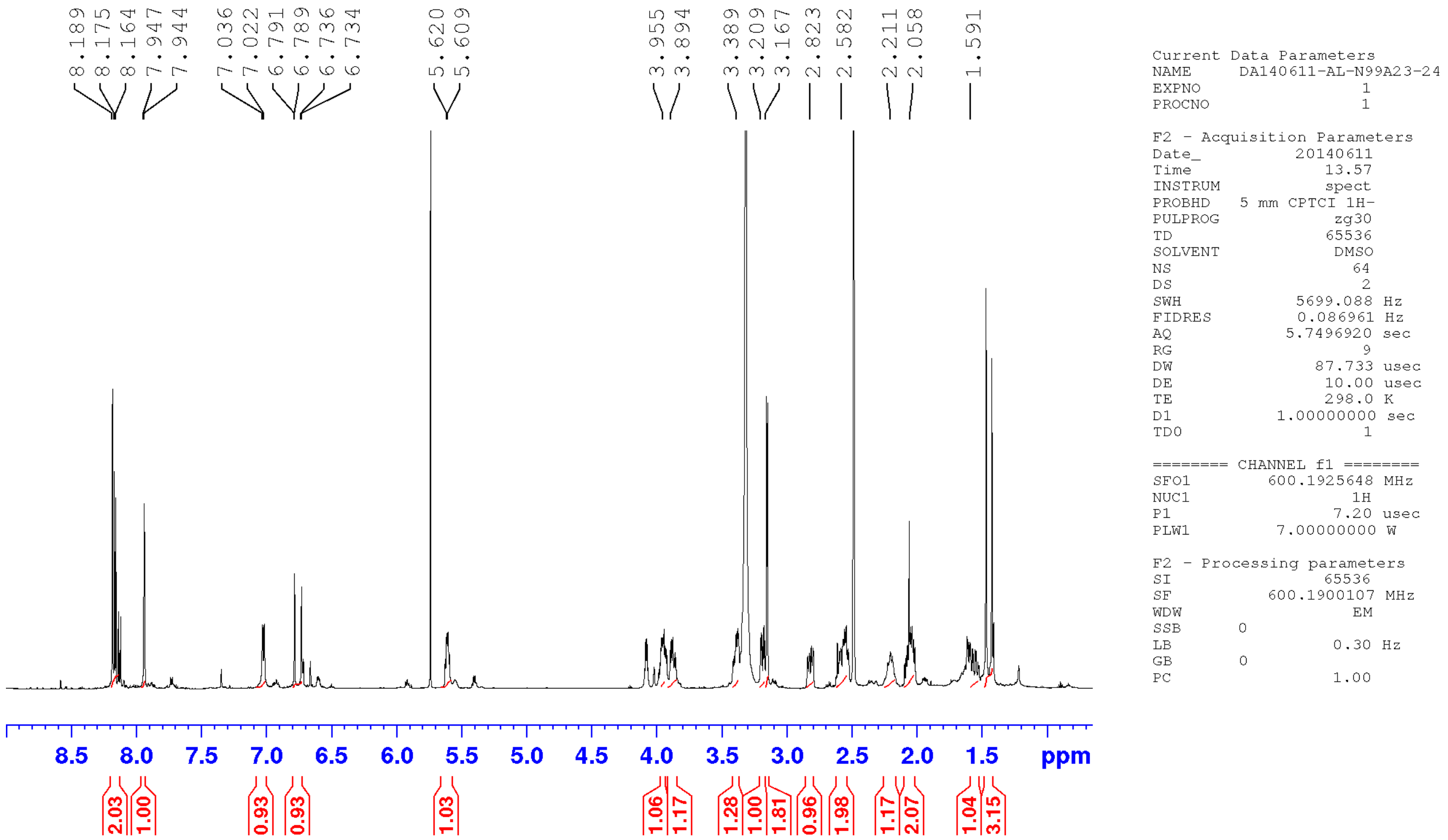

Figure S25. ^{1}H NMR spectrum of the mixture of Xestoadociaminals C (4a) and D (4c) in DMSO-d_6 (600 MHz).

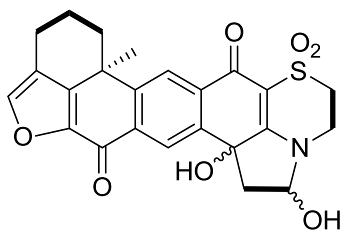

4a+4c

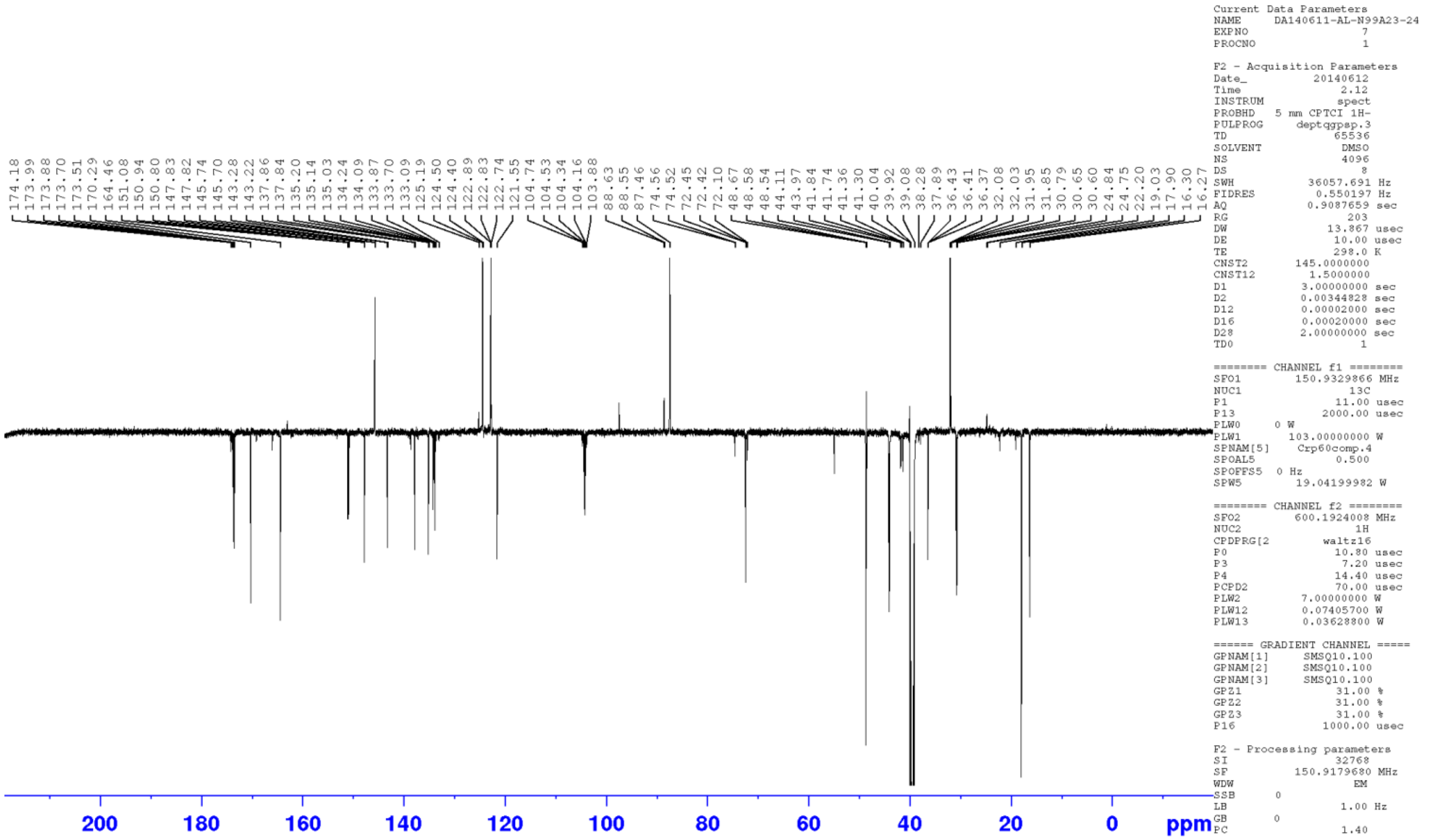

Figure S26. DEPT spectrum of the mixture of Xestoadociaminals C (4a) and D (4c) in DMSO-d_6 (150 MHz).

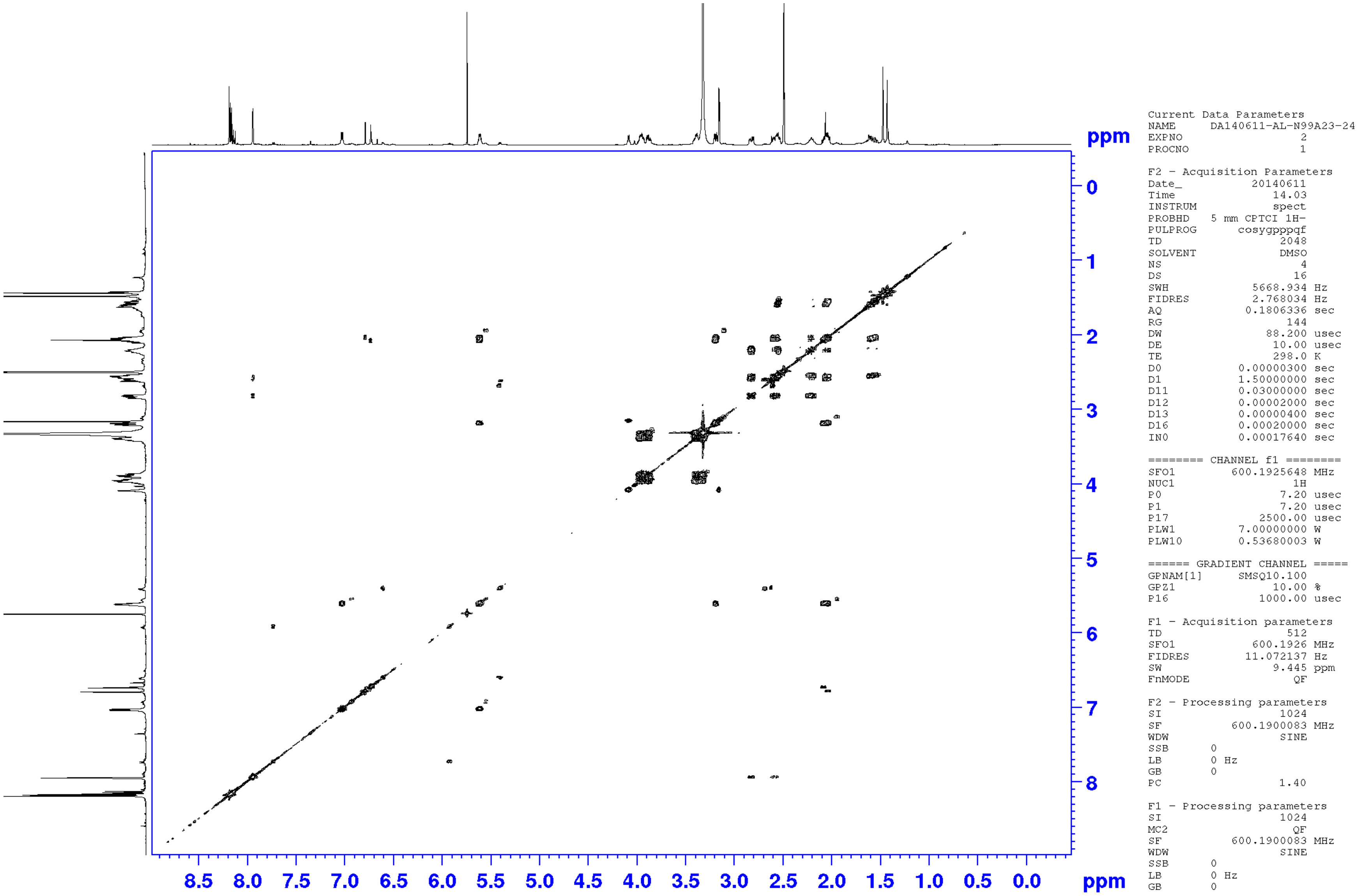

Figure S27. ^{1}H-^{1}H COSY spectrum of the mixture of Xestoadociaminals C (4a) and D (4c) in DMSO-d_6 (600 MHz).

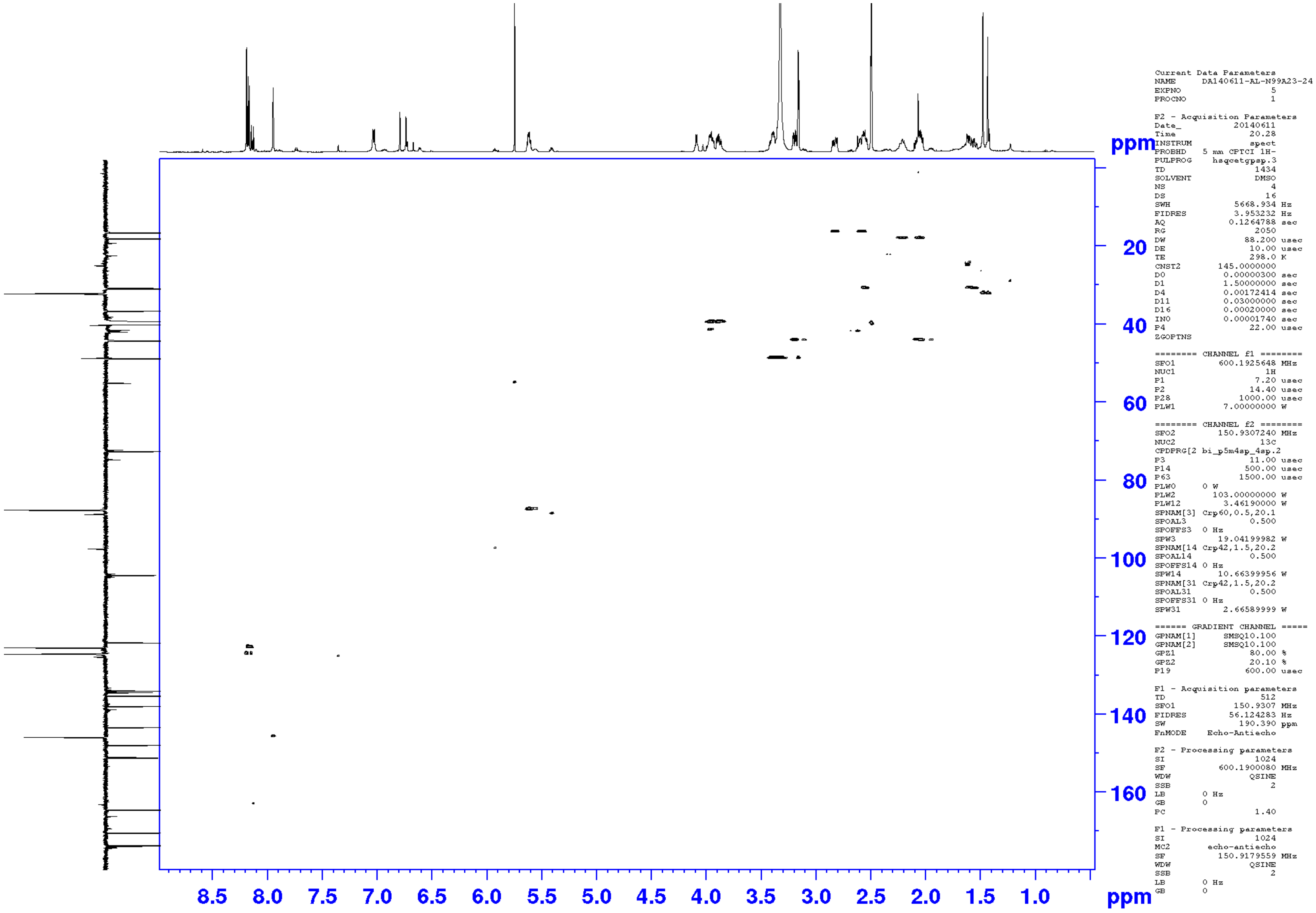

Figure S28. HSQC spectrum of the mixture of Xestoadociaminals C (4a) and D (4c) in DMSO-d_6 (600 MHz).

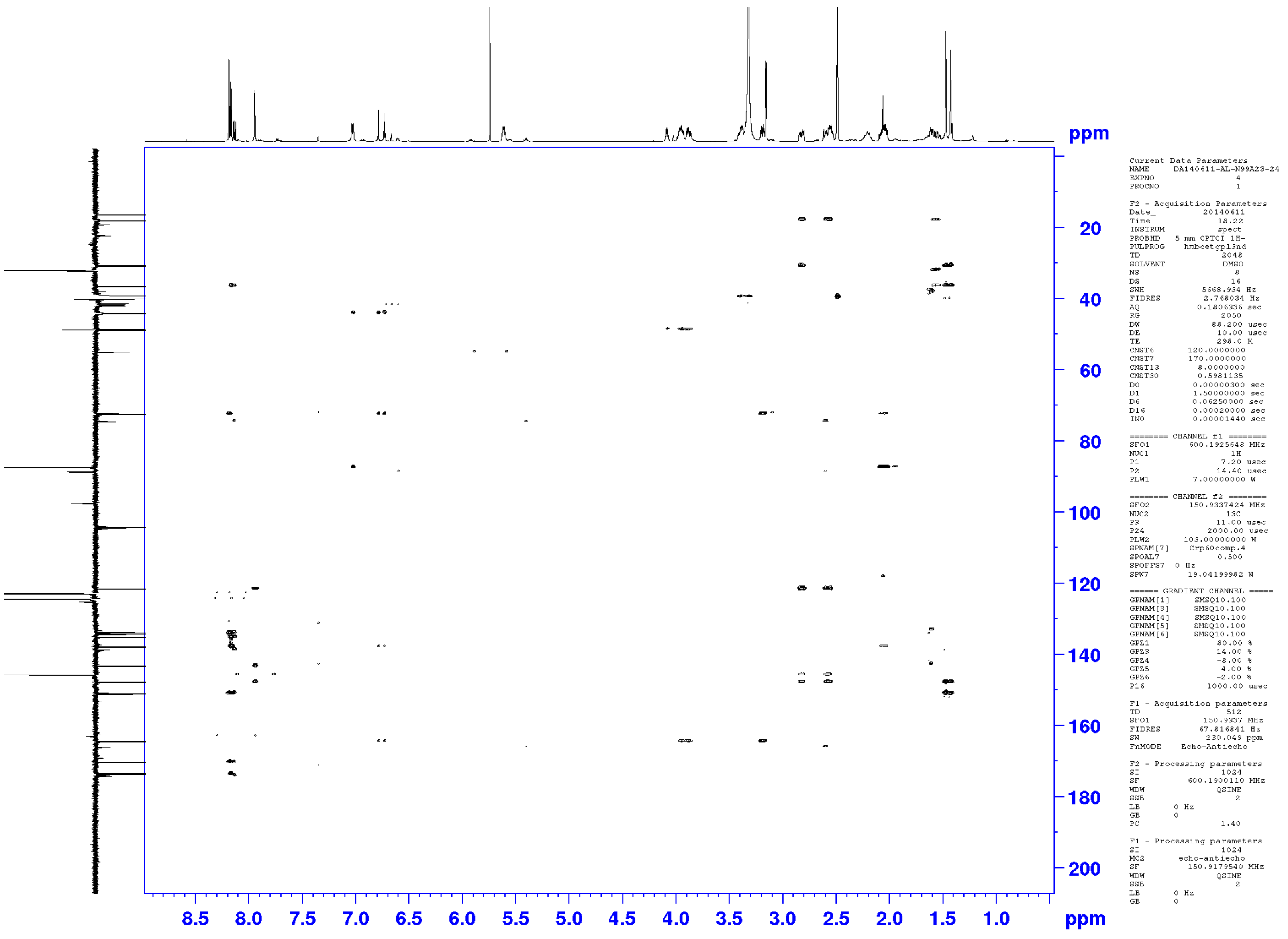

Figure S29. HMBC spectrum of the mixture of Xestoadociaminals C (4a) and D (4c) in DMSO-d_6 (600 MHz).

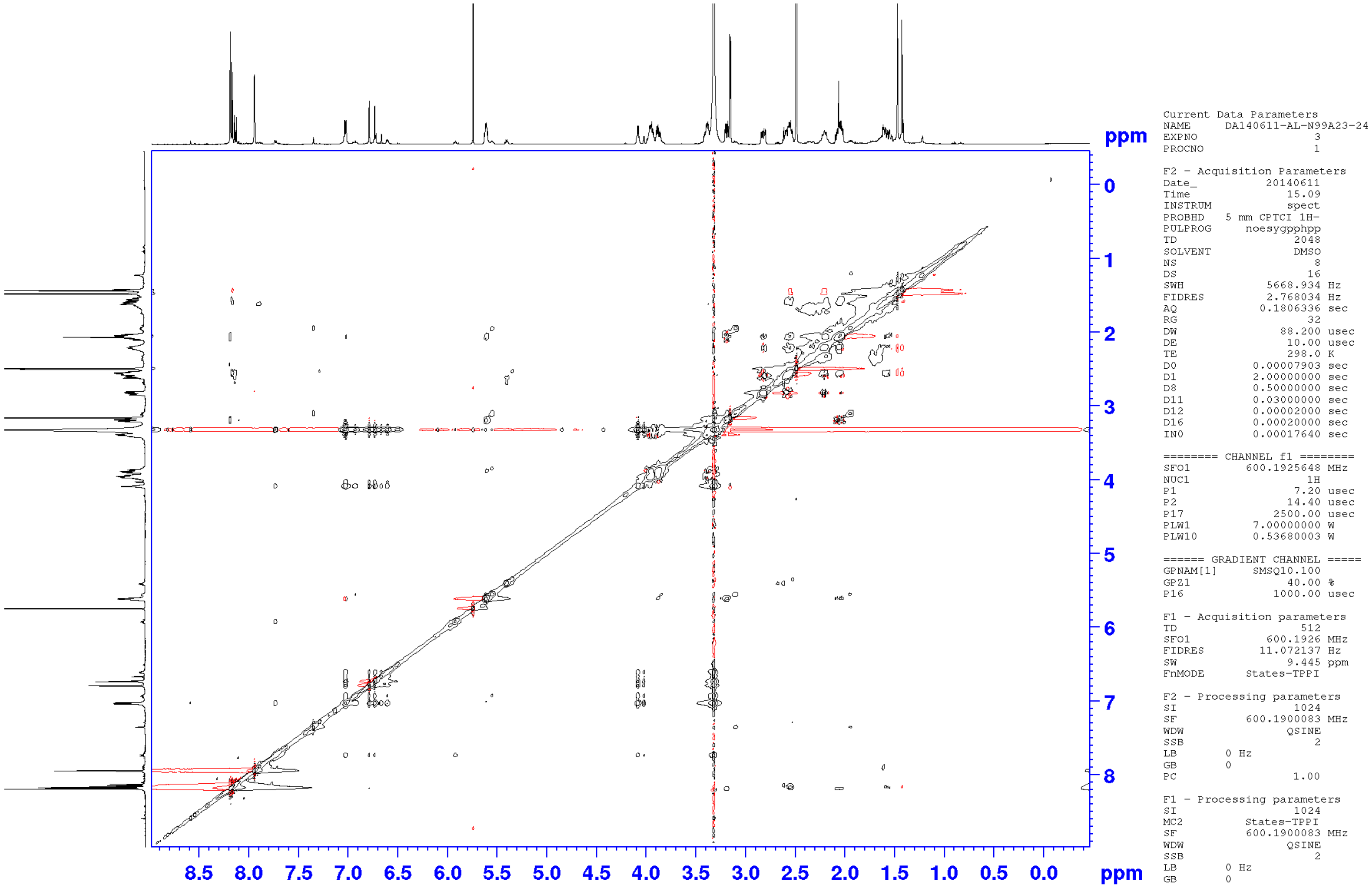

Figure S30. NOESY spectrum of the mixture of Xestoadociaminals C (4a) and D (4c) in DMSO-d_6 (600 MHz).

© 2015 by the authors; licensee MDPI, Basel, Switzerland. This article is an open access article distributed under the terms and conditions of the Creative Commons Attribution license (http://creativecommons.org/licenses/by/4.0/).
